# Dscam1 establishes the columnar units through lineage-dependent repulsion between sister neurons in the fly brain

**DOI:** 10.1038/s41467-020-17931-w

**Published:** 2020-08-13

**Authors:** Chuyan Liu, Olena Trush, Xujun Han, Miaoxing Wang, Rie Takayama, Tetsuo Yasugi, Takashi Hayashi, Makoto Sato

**Affiliations:** 1 Laboratory of Developmental Neurobiology, Graduate School of Medical Sciences, Kanazawa University, 13-1 Takaramachi, Kanazawa, Ishikawa 920-8640 Japan; 2 Mathematical Neuroscience Unit, Institute for Frontier Science Initiative, Kanazawa University, 13-1 Takaramachi, Kanazawa, Ishikawa 920-8640 Japan

**Keywords:** Developmental neurogenesis, Neural stem cells, Axon and dendritic guidance, Cell fate and cell lineage

## Abstract

The brain is organized morphologically and functionally into a columnar structure. According to the radial unit hypothesis, neurons from the same lineage form a radial unit that contributes to column formation. However, the molecular mechanisms that link neuronal lineage and column formation remain elusive. Here, we show that neurons from the same lineage project to different columns under control of Down syndrome cell adhesion molecule (Dscam) in the fly brain. Dscam1 is temporally expressed in newly born neuroblasts and is inherited by their daughter neurons. The transient transcription of *Dscam1* in neuroblasts enables the expression of the same Dscam1 splice isoform within cells of the same lineage, causing lineage-dependent repulsion. In the absence of Dscam1 function, neurons from the same lineage project to the same column. When the splice diversity of Dscam1 is reduced, column formation is significantly compromised. Thus, Dscam1 controls column formation through lineage-dependent repulsion.

## Abstract (web summary)

Columns are the functional and morphological unit of the brain, but how neurons assemble into this structure was unclear. Here, the authors show that Dscam gene rewires neurons that derive from the same stem cell to establish columns through the process of lineage-dependent repulsion.

## Introduction

Columns are the higher-order morphological and functional units of the brain. A group of neurons gather to form individual columnar units, which are then precisely arranged to establish the brain. Several types of columnar units have been described in the cerebral cortex: cortical columns are groups of cells that share similar response selectivity, and microcolumns are cell type-specific clusters of neurons^1–3^, which are found in nearly all examined cortical regions.

The radial unit hypothesis was proposed to explain the mechanism of column formation in the mammalian cerebral cortex. According to this hypothesis, columns are formed by clonally related neurons that are produced from a common progenitor cell^4^. The neurons of an individual radial unit are suggested to form a column that shares a similar response selectivity. However, sister neurons actually undergo lateral dispersion during development, become sparsely distributed and are mixed with neurons derived from other progenitors, calling into question the organization of columns simply via the clonally related neurons^5,6^. Thus, developmental mechanisms of columnar unit formation and significance of the neuronal lineage remain elusive.

**Fig. 1:**
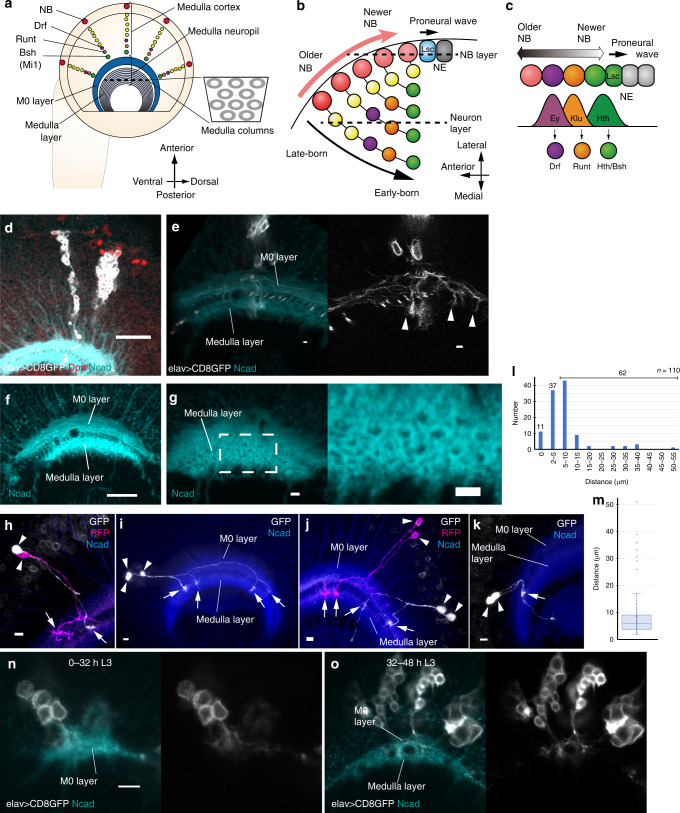
Linage dependent repulsion in the larval medulla. **a**, **b** Schematics of the developing *Drosophila* medulla in L3 larval stage. Lateral (**a**) and dorsal (**b**) views. The dotted line in (**a**) indicates the plane showing the columns in the medulla layer. The dotted lines in (**b**) indicate the planes showing the NB and neuron layers. **b**, **c** Schematics of the proneural wave and temporal transcription factors. **d**, **e** Neurons of the same lineage are visualized by *elav-Gal4* MARCM clones (GFP, white). Dpn (red) and Ncad (blue) visualize the NBs and neuropil, respectively. *n* = 26 in (**d**). *n* = 21 in (**e**). **f**, **g** The medulla structures visualized by Ncad (blue). **f** M0 and medulla layers in a lateral view. **g** The medulla columns in an anterior view. **h**–**k** Neurons of the same lineage are visualized by *drf-Gal4* twin-spot MARCM clones (GFP in white, RFP in magenta). Ncad (blue). Sister neurons reroute in M0 layer, and innervate different columns in the medulla layer. Arrows and arrowheads indicate arborizations in the medulla layer and cell bodies, respectively. **l** A histogram showing the distance between pairs of neurons on the surface of the medulla layer (*n* = 110). 0 μm indicates fused indistinguichable pairs. **m** A box plot of the distance between distinguishable 110 pairs of neurons in 38 clones found in 38 independent brain samples. The cases of 0 μm distance are not included. Center line, median; box limits, upper and lower quartiles; whiskers, 1.5× interquartile range. Median and average are 5.78 and 8.50 μm, respectively. Source data are provided as a Source Data file. **n**, **o** Projections of medulla neurons in early L3 larval stage visualized by *elav-Gal4* MARCM clones (GFP in white). Ncad (blue). **n** 0–32 h L3 larva only showing M0 layer (*n* = 20). Neurites are already defasciculated within M0 layer. **o** 32–48 h L3 larva showing M0 and medulla layers (*n* = 49). Neurites are defasciculated in M0 layers and innervate the medulla layer. **d**–**f**, **h**–**k**, **n**, **o** Lateral views showing the neuron layer in (**b**). **g** Anterior view showing the columns along the dotted line in (**a**). Scale bars indicate 20 μm in (**d**, **f**, **n**, **o**) and 5 μm in (**e**, **g**, **h**–**k**).

Like the mammalian brain, the fly visual system shows columnar organizations such as ommatidia in the retina, cartridges in the lamina, and columns in the medulla^7–10^. Photoreceptor neurons R1–8 form an ommatidium in the retina, while R1–6 neurons and lamina neurons L1–5 form a cartridge in the lamina. The medulla is the largest component of the fly visual system and each medulla column contains as many as 100 neurons. Medulla neurons make connections and form columnar units in the medulla neuropil^8^. Note that medulla neurons, whose cell bodies are situated in the medulla cortex, project their neurites toward the medulla neuropil (Fig. 1a). The axons and dendrites of neurons form repetitive columnar units in the medulla neuropil.

In our previous study, we demonstrated that R7, R8, and Mi1 are the core neurons that are concentrically arranged in the larval medulla according to N-cadherin-dependent differential adhesion^7^. Compared with the retina and lamina, developmental sequence of the medulla is more similar to that in the cerebral cortex. A single neuroblast (NB; a neural stem-like cell), produces a group of radially oriented and clonally related neurons that is analogous to the radial unit, a group of neurons that is produced from a single neural progenitor cell and migrates along the same radial fiber in the developing cerebral cortex (Fig. 1a)^11,12^. The development of ommatidia and lamina cartridges does not accompany NB and lineage-dependent development.

In a previous study, we demonstrated that the wave of differentiation known as the proneural wave sweeps across the sheet of neuroepithelial cells (NEs) in the developing larval optic lobe. NEs are sequentially differentiated into medulla NBs behind the proneural wave (Fig. 1b)^13–15^. Thus, NBs are arranged according to their birth order and sequentially produce different types of neurons inside the brain. We and other groups have demonstrated that temporal transcription factors, such as Homothorax (Hth), Klumpfuss (Klu), and Eyeless (Ey), which are sequentially expressed in NBs, specify birth order-dependent production of neurons (Fig. 1c). Each NB and its daughter neurons form a radially arranged cluster of neurons during larval stage (Fig. 1a). The birth order of medulla neurons correlates with the concentric gene expression found in the medulla cortex. For example, Hth-positive NBs produce neurons that inherit Hth expression, which in turn induces Brain-spefic-homeobox (Bsh) expression. As a result, newly differentiated NBs produce Hth/Bsh double-positive Mi1 neurons that are located in the inner most concentric domain in the medulla cortex (Fig. 1a)^16,17^. Similarly, Klu- and Ey-positive NBs produce Runt- and Drifter (Drf)-positive neurons, respectively, forming the concentric domains outside of the Hth/Bsh domain^11,16,17^. Similar temporal patterning of neural stem cells and neurogenesis are found in the developing cerebral cortex^12^.

During larval stage, medulla neurons of the same lineage are radially arranged, forming a radial cluster. However, these neurons are tangentially dispersed in pupal stage. As a result, the neurons of the same lineage are no longer clustered anymore beyond 24 h after puparium formation (APF)^11,18,19^. This phenomenon is similar to rather sparse distribution of the sister neurons found in the mature cerebral cortex^5,6^. Thus, columns are not simply formed according to the radial unit of the same neuronal lineage in the fly medulla and mammalian cerebral cortex.

In this study, we demonstrate that sister neurons of a given lineage project to different columns in the larval medulla, suggesting that the neurons of the same lineage repel each other, which we refer to as lineage-dependent repulsion. As a potential candidate molecule that could regulate this process, we focus on *Down syndrome cell adhesion molecule* (*Dscam*), a gene significantly contributing to the phenotypes observed in Down syndrome^20,21^.

*Drosophila Dscam1* gene has three alternative exons encoding Ig2, Ig3, and Ig7 domains containing 12, 48 and 33 different splice variants, respectively. In total, *Dscam1* encodes as many as 19,008 different ectodomains^22^. Homophilic binding of Dscam1 only occurs between identical isoforms that match at all three variable Ig domains and produces a repulsive signal^23^. Thus, neurons expressing the same Dscam1 isoforms show a repellent interaction. In addition, splicing of *Dscam1* in each cell is probabilistic^24^. The vast diversity of Dscam1 isoforms is necessary for correct development of neural circuits^25^. Expression of the same Dscam1 isoform in a single cell causes self-avoidance, which is important for correct dendritic wiring^26^.

We hypothesize that Dscam1 may be temporally expressed in NBs and is inherited by neurons of the same lineage to regulate the lineage-dependent repulsion. Indeed, we show that Dscam1 is temporally expressed in NBs under the control of Hth, a temporal transcription factor. Expression of Dscam1 in a radial unit is essential for lineage-dependent repulsion. Our findings suggest a function of Dscam1 in lineage-dependent repulsion, which provides a link between temporal patterning, neuronal lineage and column formation.

## Results

### Lineage-dependent repulsion in the developing medulla

NBs are located in the outermost region of the larval medulla primordium and produce a group of neurons toward the inner area of the medulla cortex with a radial orientation, as visualized by GFP expressed under the control of *elav-Gal4* using the MARCM technique in order to label neurons of the same lineage (Fig. 1a, d). Daughter neurons of the same NB are linearly arranged in the larval brain forming a radial unit until the onset of tangential dispersion between 12 and 24 h APF^11^. By closely focusing on their neurites, we found that neurons of the same lineage widely project their axons encompassing multiple columns (Fig. 1e). During the late 3rd larval stage (L3), the developing neuropil, as visualized with the Ncad antibody, contains two distinct layers (Fig. 1e, f). The medulla layer contributes to adult medulla layers M1–M10. The other layer, located outside the medulla layer, is a temporal layer that disappears during the pupal stage^11^. We refer to this temporal structure as M0 layer (Fig. 1a, e, f). The medulla columns can be observed within the medulla layer in a frontal view (Fig. 1a, g). Note that the distance between neighboring columns is ~5 µm, while neurites of a radial unit extend as far as 50 µm distance and more (Fig. 1e, g; *n* = 8).

The axons change their directions within M0 layer and eventually project to the medulla layer (Fig. 1e). To clearly distinguish each of the axons, we used twin-spot MARCM technique under the control of *drf-Gal4*, which visualizes a smaller number of neurons (Fig. 1h–k)^27^. In many cases, the axons change their direction within M0 layer, and project to the medulla layer or to the other brain region such as lobula through the medulla layer, which is reminiscent of the projection patterns in Tm-type neurons^11,28^.

Usually, sister neurons that derive from the same NB do not form projections to the same columns. Instead, they are often rerouted in M0 layer and form projections to different columns within different regions of the medulla layer (Fig. 1h–j). Within the medulla cortex, axons of the same radial unit are bundled and project together toward M0 layer. However, they are defasciculated within M0 layer and project to different columns in the medulla layer.

We quantified the distance between sister neurons that derive from the same radial unit by focusing on brain samples that contain small number of isolated clones (Fig. 1l, m). When the axons were fused, we regarded the distance as 0 μm. Otherwise, the distance between axon shafts on the surface of the medulla layer was measured (Fig. 1l). According to Ncad staining, the distance between columns is about 5 μm in larval medulla (Fig. 1g). Among 110 pairs of neurons, 11 and 37 pairs were 0 μm and 2–5 μm distant, respectively, and 62 pairs projected to the medula layer showing distance >5 μm (Fig. 1l). Note that 2–5 μm distance does not necessarily mean that they are projecting to the same column, because they may still project to the adjacent domains of the neighboring columns.

Distribution of the distance between axon pairs is plotted in Fig. 1m. Median and average of the distance are 5.78 and 8.50 μm, respectively. The behavior of the axons suggests that the sister neurons repel each other very often. Since this repulsion occurs between neurons of the same lineage, we refer to this process as lineage-dependent repulsion.

When we focused on the anterior part of the developing medulla, neurons of the same lineage often projected to distinct parts of the medulla neuropil (*n* = 27/32; Fig. 1h–j). However, in the posterior part of the medulla, the terminals of the sister neurons were often indistinguishable (*n* = 8/24; Fig. 1k). Among 11 pairs of fused axons, 9 were located in the posterior part of the medulla (Fig. 1l). Thus, lineage-dependent repulsion may be less prominent in the posterior part of the medulla. In the followings sections, we only focus on the anterior part of the medulla.

To examine when lineage-dependent repulsion takes place early in development, we examined 0–32 h and 32–48 h L3 larval brains (Fig. 1n, o). In 0–32 h L3 brains, there was only one Ncad-positive layer, which is most likely M0 layer, because all axons project through M0 layer. The medulla layer is then found in 32–48 h L3 brains. Importantly, the axons of the same lineage are already defasciculated within M0 layer in the 0–32 h L3 stage. Thus, lineage-dependent repulsion takes place even before the formation of the medulla layer (Fig. 1n).

**Fig. 2:**
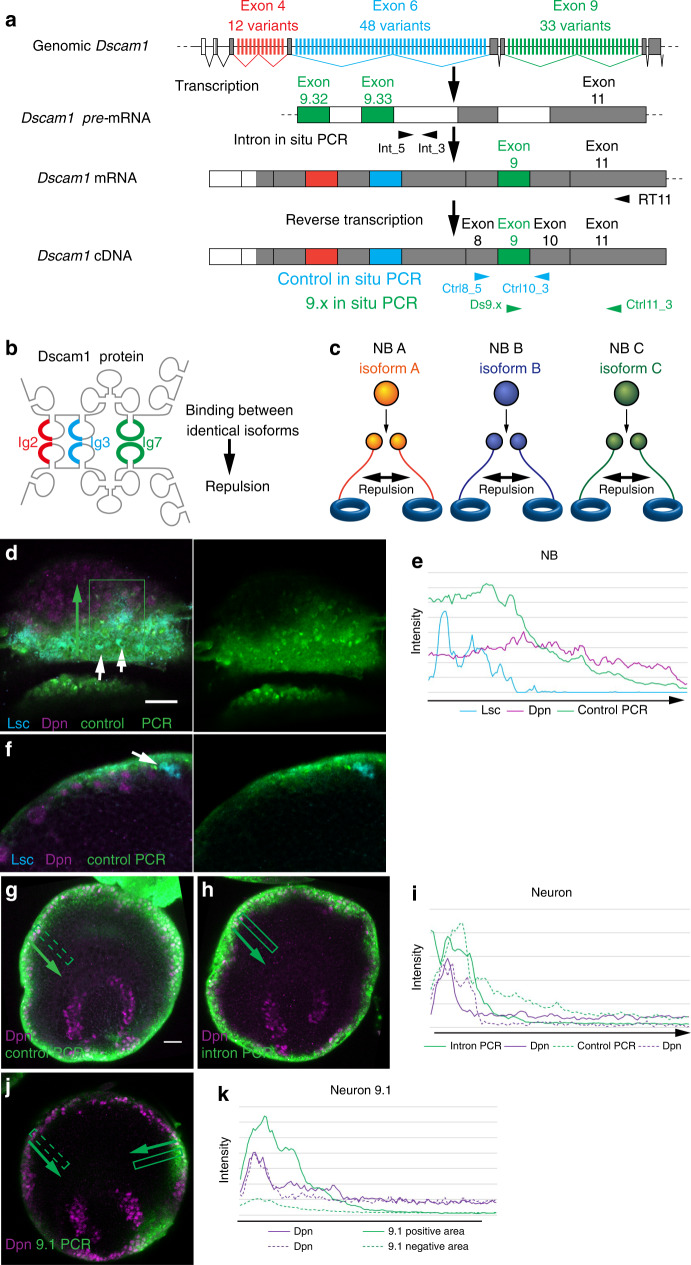
Detecting transcription of Dscam1 in NB and neurons using in situ RT-PCR. **a** Schematic of *Dscam1* gene structure and alternative splicing indicating the primers used for in situ RT-PCR. **b** Homophilic binding of the identical Dscam1 isoform causes repulsion. **c** Schematic representation of lineage depend repulsion between neurons that derive from the same NB expressing the same Dscam1 isoform. **d** Control PCR (green), Lsc (blue) and Dpn (magenta) on the surface of the brain in a lateral view showing the NB layer, *n* = 22 (see Fig. 1b). **e** Quantification of signal intensity in the dotted box in (**d**). **f** Control PCR (green), Lsc (blue) and Dpn (magenta) in a dorsal view showing the decrease of mRNA signal in older NBs, *n* = 16 (same orientation as Fig. 1b). **g**, **h**, **j** Lateral views showing the neuron layer (see Fig. 1b). **g** Control PCR (green) and Dpn (magenta), *n* = 47. **h** Intron PCR (green) and Dpn (magenta), *n* = 13. **i** Quantification of signal intensity in the boxes in (**g**, **h**). Background signal was subtracted for Dpn. **j***Dscam1* 9.1 PCR (green) and Dpn (magenta) in a lateral view showing the neuron layer, *n* = 10 (see Fig. 1b). **k** Quantification of signal intensity in the boxes in (**j**). Each box contains one NB. Intensities in the dotted boxes are plotted with dotted lines. Scale bars indicate 20 μm. Source data are provided as a Source Data file (**e**, **i**, **k**).

To enable lineage-dependent repulsion, daughter neurons that derive from the same NB must remember the identity of their common mother NB and repeal each other according to their lineage. *Dscam1* potentially exhibits nearly 20,000 splice variants (Fig. 2a). Identical Dscam1 isoforms bind with each other and provides a repulsive signal (Fig. 2b). Self-avoidance of dendritic processes is controlled by the same Dscam1 isoform expressed in the same neuron^26^. A similar mechanism may regulate lineage-dependent repulsion in the medulla column. However, in this case, repulsion must occur between a group of neurons that derive from the same NB. Since splicing diversity of Dscam1 is thought to be stochastically selected^24^, we assume that each NB temporally expresses a single Dscam1 variant, which is inherited by its daughter neurons. Therefore, the daughter neurons that are produced by the same NB likely express the same Dscam1 variant and repel each other, leading to projection to different medulla columns (Fig. 2c). In contrast, neurons of different lineages expressing different variants do not repel each other and are able to project to the same column.

### Transcription of *Dscam1* essentially occurs in NEs and NBs

To test this hypothesis, transcription pattern of *Dscam1* in NBs and neurons was examined. To detect low levels of mRNA, reverse-transcribed cDNA was PCR-amplified to perform in situ RT-PCR for *Dscam1* mRNA (Fig. 2a; “Methods”). We designed control primers that amplify a fragment containing exons 8–10, which is shared by all *Dscam1* isoforms (Fig. 2a).

During larval development, NEs sequentially become NBs in a medial-to-lateral orientation on the surface of the developing medulla behind the proneural wave (Fig. 1b). Lsc is transiently expressed in a narrow band of 1–2 NE cells at the wavefront^15^, whereas Dpn is strongly expressed in all NBs. Strong mRNA signals were found in NEs and NBs following the wavefront of the proneural wave, as indicated by Lsc expression and gradually decreased in the older NBs (Fig. 2d–f), suggesting that Dscam1 is temporally transcribed in the newborn NBs. The signals decreased as the neurons became older in the inner part of the brain (Fig. 2g, i). The observation that strong *Dscam1* mRNA signals form a circle encompassing the entire larval brain hemisphere (Fig. 2g, Supplementary Fig. 1a) indicates that they are temporally transcribed in all of the newborn medulla NBs and inherited to their daughter neurons.

To confirm whether *Dscam1* is newly transcribed in the medulla neurons, or not, we detected pre-mRNA of *Dscam1* by using a primer set that amplifies the intron between the exons 9.33 and 10 (Fig. 2a). As we expected, the intron PCR signals were highly restricted to NBs on the surface of the brain hemisphere (Fig. 2h, i). The signals in neurons were hardly detectable compared with control RT-PCR results (Fig. 2i). These results suggest that *Dscam1* mRNA is essentially transcribed in NEs and newborn NBs immediately behind the proneural wave, and is inherited to the daughter neurons.

To confirm the validity of our in situ RT-PCR technique, we examined *Dscam1* mRNA signal in clones homozygous for *Dscam*^*20*^, a null mutant of *Dscam1* (Supplementary Fig. 1b). Compared with control cells, signals for Dscam1 protein and *Dscam1* mRNA as visualized by in situ RT-PCR were abolished, suggesting that in situ RT-PCR specifically detects *Dscam1* mRNA.

We also visualized *Ncad* mRNA by using in situ RT-PCR (Supplementary Fig. 1c–e). Consistent with Ncad protein expression in medulla neurons, we observed strong *Ncad* mRNA signal in the inner region of the medulla cortex. Relatively uniform *Ncad* mRNA signal throughout the medulla strongly suggests that the sharp decrease of *Dscam1* mRNA signal inside the brain indeed recapitulates *Dscam1* expression (Fig. 2g–i).

### Neurons of the same lineage express similar Dscam1 isoforms

To test the hypothesis that the same *Dscam1* isoform is inherited by the daughter neurons of a NB, we performed in situ RT-PCR for a single variant of exons 4, 6, and 9 (Fig. 2a and Supplementary Fig. 1f–k). According to the results of the previous study, one splice variant is stochastically chosen from the alternative exon 4^24^. If the same phenomenon occurs for exons 6 and 9, a limited number of medulla NBs should express the same exon variant, which will then be inherited by their daughter neurons. Indeed, we observed a cluster of NBs and their daughter neurons expressing the same variant of exons 4, 6 and 9 (Fig. 2j, k and Supplementary Fig. 1f–n). The location of 9.1-positive NB cluster was not uniform, but variable in each brain sample. In many cases, a brain contained one or two domains that express a particular exon variant. We repeated the same experiment for 22 splice variants from exon 4 (4 variants), 6 (10 variants) and 9 (8 variants; “Methods”). At least ten samples were observed for each variant, and we obtained essentially the same results as quantified in Supplementary Fig. 1l–n, suggesting that an alternative splice variant is stochastically chosen.

If the stochastic choise solely occurs in NBs, a salt-and-pepper-like pattern should appear. Indeed, closer look at their expression patterns occasionally reveals a lack of in situ RT-PCR signals in an expression domain (Supplementary Fig. 1i). However, this may be due to cell cycle dependent changes in mRNA distribution. Since Dscam1 expression is initiated in NEs (Fig. 2d–f), which quickly divide symmetrically, a group of NBs is supposed to share the same exon variant. Or, there might be unknown mechanisms that provide a bias on the choise of alternative exons.

We assume that alternative splicing of all of the alternative exons (exons 4, 6, and 9) is independently and stochastically determined according to the previous study^24^. If so, a cluster of NBs expressing the same variant of exon 9 most likely contains NBs expressing different variants of the other exons and can presumably be further subdivided by the selection of exons 4 and 6. Thus, one or very small number of NB lineages could share exactly the same splice variants.

On the other hand, we also expressed a single isoform of Dscam1 in neurons by generating clones of cells containing a single Dscam1 isoform (*dscam*^*3.31.8*^; Supplementary Fig. 2a–e). The neurons expressing the single isoform showed a normal radial arrangement, and their neurites were normally defasciculated in M0 layer projecting to the wide area of the medulla neuropil, as found in wild-type control clones (Supplementary Fig. 2e).

In wild-type condition, we assume that exon 9.8 is stochastically chosen upon alternative splicing. In contrast, the single-isoform mutant, *dscam*^*3.31.8*^, lacks all variants of exon 9 except for 9.8. We next asked what happens to the expression pattern of exon 9.8 in this single variant mutant background. Surprisingly, mRNA for exon 9.8 was uniformly detected in the medulla NBs forming a circle encompassing the entire larval brain hemisphere (Supplementary Fig. 2f, g), suggesting that exon 9.8 is always chosen in the absence of the other variants of exon 9. Consistently, mRNA for exons 9.1 and 9.4 were not detected in the same mutant background (Supplementary Fig. 2h, i). These findings support our hypothesis that neurons of same lineage express similar Dscam1 isoforms.

### Dscam1 protein is stabilized in medulla neurons

**Fig. 3:**
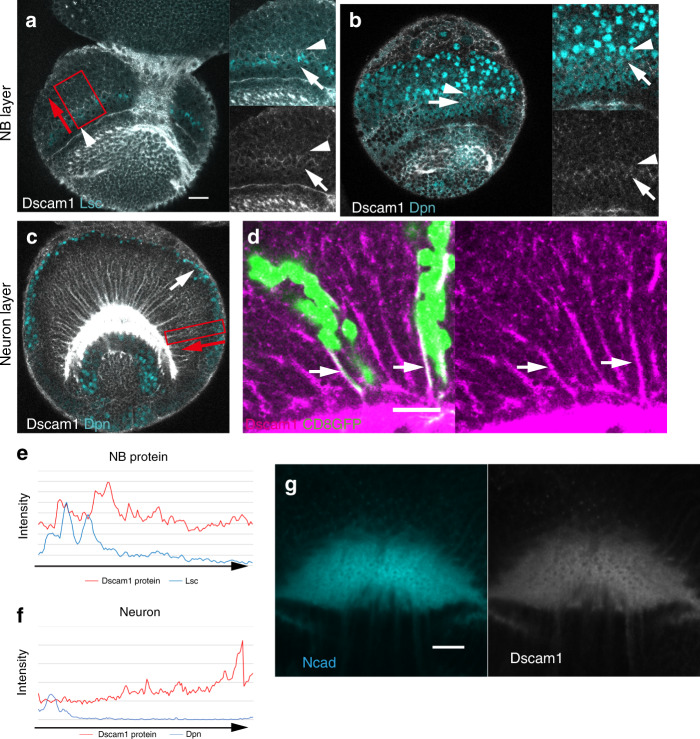
Expression pattern of Dscam1 protein. **a**, **b** Expression pattern of Dscam1 protein (white) in NEs and NBs in a lateral view showing the NB layer (see Fig. 1b). (**a**) Lsc (blue). (**b**) Dpn (blue). **c**, **d** Expression pattern of Dscam1 protein (white or magenta) in neurons in a lateral view showing the neuron layer. **c** Dpn (blue). **d** Arrows indicate Dscam1 signals along the axons of GFP expressing neurons (*Ay-Gal4 UAS-GFP*; green). **e**, **f** Quantification of Dscam1 signal intensity in the boxes in (**a**) and (**c**), respectively. **g** Dscam1 (white) and Ncad (blue) visualize the columnar structures in the medulla layer in an anterior view (dotted line in Fig. 1a). Scale bars indicate 20 μm. Source data are provided as a Source Data file (**e**, **f**).

Next, we examined the expression pattern of the Dscam1 protein in NBs and neurons. We found that Dscam1 protein is weakly expressed in Lsc-positive NEs and 1–2 rows of Dpn-positive NBs behind the proneural wavefront and decreases in older NBs (Fig. 3a, b, e), suggesting that Dscam1 protein is temporally expressed accompanying NB differentiation, but is rapidly downregulated in older NBs.

In contrast to mRNA distribution, Dscam1 protein is strongly accumulated along the neural fibers that are radially oriented in the medulla cortex, which colocalize with neurites projecting from the radial cluster of neurons (Fig. 3c, d, f). Since Dscam1 signals in NBs and neurons are eliminated in *Dscam1* null mutant clones (*dscam1*^*20*^; Supplementary Fig. 1b), the above signals indeed reflect the expression patterns of Dscam1. Thus, we assume that *Dscam1* is predominantly transcribed in newborn NBs behind the proneural wave, while *Dscam1* mRNA inherited by their daughter neurons is rapidly degraded. On the other hand, Dscam1 protein, which may be translated in NBs and neurons, is stabilized in neurons and localizes to the neurites (Fig. 3d).

The temporal restriction of *Dscam1* transcription may be essential for lineage-dependent repulsion. During alternative splicing, spliceosome machinery assembles at the splice sites forming a complex that leads to the selection of a single splice variant^29^. If the duration of transcription is restricted, a small number of splice variants will be selected. As a result, a NB will produce a single or very small number of splice isoforms, which are shared among its daughter neurons. When a group of neurons expresses the identical Dscam1 isoform, the recognition between Dscam1 proteins causes mutual repulsion, leading to lineage-dependent repulsion (Fig. 2b, c).

The strong Dscam1 signals found in M0 layer (Fig. 3c, d) are consistent with the idea that Dscam1 regulates the spreading of neurites within M0 layer in the larval medulla (Fig. 1e, h–j). The columnar distribution pattern of Dscam1 in the medulla layer, which overlaps with the columnar distribution of Ncad, also suggests its essential role in column formation (Fig. 3g).

### Hth regulates temporal Dscam1 expression in NBs

Similar to Dscam1, expression of Hth and Ey in NBs is inherited by the daughter neurons in the larval medulla^11,16,17^. Hth is the first temporal transcription factor expressed in NEs and NBs. Hth activates expression of Bsh and Ncad in the early born medulla neurons, which differentiate to a single type of medulla neuron, Mi1 (Fig. 1c)^11,30^.

**Fig. 4:**
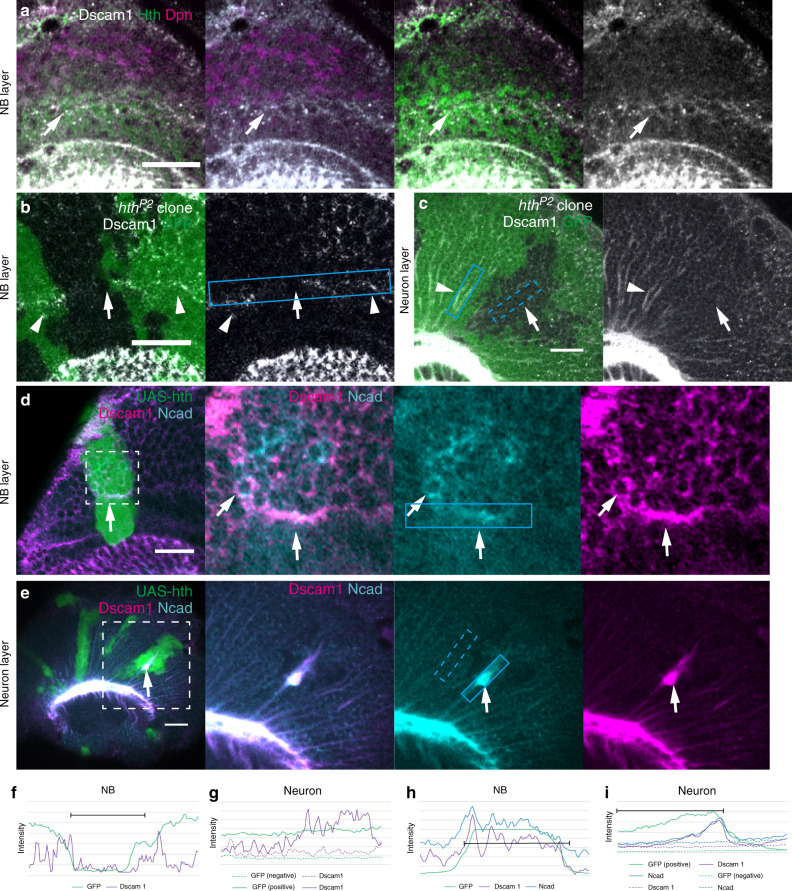
Hth regulates the expression of Dscam1. Lateral views of L3 larval brains showing the NB (**a**, **b**, **d**) and neuron layers (**c**, **e**; see Fig. 1b). Dscam1 (white in **a**–**c**, magenta in **d**, **e**). **a** Dscam1 expression overlaps with Hth (green) in NEs, and with Hth and Dpn (magenta) in NBs. **b**, **c** Background level of Dscam1 signal in *hth* mutant clones visualized by the absence of GFP (green; arrows) compared with the control cells (arrowheads). Pixel intensity of Dscam1 signal was uniformly enhanced throughout the image (**b**). **d**, **e** Ectopic Dscam1 upregulation in clones expressing *hth* visualized by GFP (green; arrows) under the control of *Ay-Gal4*. Ncad (blue). The areas indicated by the dotted boxes are enlarged in the right panels. Scale bars indicate 20 μm. **f**–**i** Quantification of signal intensity in the boxes in (**b**–**e**). Intensities in the dotted boxes are plotted with dotted lines. **f**, **g** Dscam1 signal is reduced in GFP-negative *hth* mutant clones (**b**, **c**). **h**, **i** Dscam1 signal is enhanced in GFP-positive *hth* expressing clones (**d**, **e**). Background signal was subtracted for Dscam1 (**f**, **h**). Experiment was independently repeated at least three times with similar results (**a**–**e**). Source data are provided as a Source Data file (**e**, **g**, **h**, **i**).

We compared the expression patterns of Dscam1, Hth, Bsh, and Ncad, and found that Dscam1 and Hth are coexpressed in NEs and newborn NBs (Fig. 4a). In contrast, Bsh and Ncad are specifically expressed in neurons and not in NEs/NBs^11,30^. Strong Dscam1 signals were detected in neurons located in the inner area of the developing medulla (Fig. 3c, d). Similarly, a transcriptional regulator, Engrailed, regulates the expression of a guidance receptor, Frazzled, in NBs to control axon guidance during *Drosophila* embryonic development^31^.

Since Hth is a temporal transcription factor whose expression in NEs and NBs overlaps with that of Dscam1 and activates the expression of Bsh and Ncad in neurons, it may also regulate the expression of Dscam1. To test this possibility, we generated *hth* mutant clones. As expected, Dscam1 expression in the medulla NEs and NBs was autonomously eliminated in *hth* mutant clones (17/43; Fig. 4b, f). The Dscam1 signals in medulla neurons were reduced (Fig. 4c, g). The residual Dscam1 signals may be due to nonspecific background of Dscam1 antibody; because similar background signals were also detectable in *Dscam1* null mutant clones (Supplementary Fig. 3b). The strong Dscam1 signals along the neurites were completely eliminated in *hth* mutant clones (10/41; Figs. 3c, d and 4c, g).

Note that the *hth* mutant used in this study (*hth*^*P2*^) is the most commonly used allele, but is hypomorphic (Fly Base). The incomplete loss of Dscam1 expression may be due to its hypomorphic nature. Or, there might be additional unknown factors that act partially redundantly with *hth*.

To test whether *hth* expression is sufficient to induce Dscam1 expression, we generated clones ectopically expressing *hth* (Fig. 4d, e). We found that ectopic *hth* expression in NBs effectively upregulated the expression of Dscam1 (11/21; Fig. 4d, h). In addition, Dscam1 expression was upregulated in neurons and localized along the neurites upon ectopic *hth* expression (21/47; Fig. 4e, i). The upregulation of Ncad found in *hth* expressing clones suggest that the ectopic *hth* expression causes premature neuron differentiation, which may indirectly upregulate Dscam1 (Fig. 4e). However, Dpn-positive NBs also show upregulation of Dscam1 expression on the surface of the brain (Supplementary Fig. 4). Taken together, these results indicate that Hth acts as a trigger of Dscam1 expression in NEs and NBs.

While Hth is widely expressed in NEs, Dscam1 expression is found in a part of NEs (Figs. 2d–f and 3a). Thus, Hth expression in NEs may not be sufficient to induce Dscam1 expression. The other unknown factor might be necessary to cause the full induction of Dscam1 expression.

### Loss of Dscam1 causes the loss of neural defasciculation

**Fig. 5:**
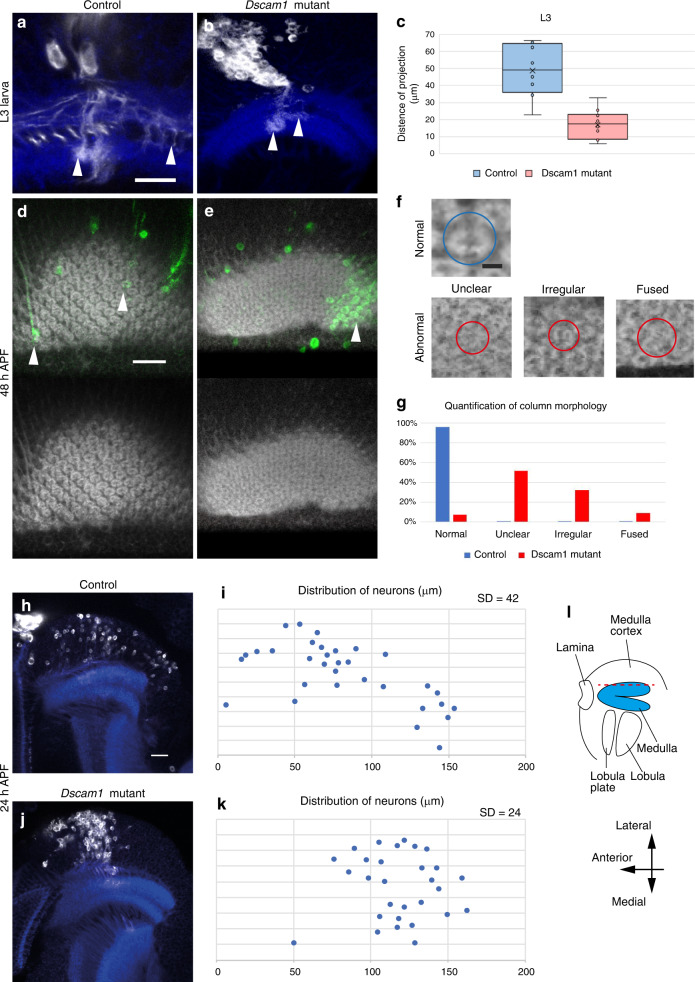
Loss of Dscam1 impairs lineage-dependent repulsion. Neurons of the same lineage are visualized by *elav-Gal4* MARCM clones (GFP in white). Ncad (blue) visualizes the neuropil structure and columns. Projection patterns of neurons of the same lineage in control (**a**) and *Dscam1* mutant clones (**b**). Lateral views of L3 larval brains showing the neuron layer (see Fig. 1b). Wide spread tangential projections found in M0 layer in control clones (**a**) are suppressed in *Dscam1* clones (**b**; arrowheads). The neurons innervate the medulla layer following M0 layer. **c** Quantification of the distance between neurites of the same lineage. Control: *n* = 8. *Dscam1* mutant: *n* = 10, Center line, median; box limits, upper and lower quartiles; whiskers, 1.5× interquartile range. Average projection distances are 49.2 and 17.45 μm, respectively. SD are 14.8 and 8.09, respectively (two-sided *t* test, *P* = 0.00043). **d**, **e** Column morphology of 48 h APF pupal medulla showing the M1–2 layers. Control (**d**) and *Dscam1* mutant clones (**e**). Regular column morphology is disrupted in and around *Dscam1* mutant clones (arrows). **f** Classification of column morphology. In contrast to normal columns, abnormal columns are classified into irregular, fused and unclear columns. **g** Quantification of column morphology. Control: 87 columns from 3 brains. *Dscam1* mutant clones: 136 columns from 3 brains (Fisher exact test, normal *P* = 8.4 × 10^−47^, unclear *P* = 7.23 × 10^−19^, irregular *P* = 7.18 × 10^−12^, fused *P* = 0.00046). **h**, **j** Tangential migration of neuronal cell bodies of the same lineage at 24 h APF. Dorsal views showing control (**h**) and *Dscam1* mutant clones (**j**). **i**, **k** Spatial distribution of the cell bodies in the medulla cortex in (**h**, **j**) are quantified. The wide spread tangential distribution in control (**h**, **i**; SD = 42 μm, *n* = 3) is suppressed in *Dscam1* mutant clones (**j**, **k**; SD = 24 μm, *n* = 3, two-sided *t* test, *P* = 0.0022). **l** Schematic representation of the optic lobe at 24 h APF. Scale bars indicate 20 μm in (**a**, **b**, **d**, **e**, **h**, **j**) and 5 μm in (**f**). Experiment was independently repeated at least three times with similar results (**a**, **b**, **d**, **e**). Source data are provided as a Source Data file (**c**, **g**, **i**, **k**).

Dscam1 protein is expressed in NBs and their daughter neurons. As we demonstrated, neurites of sister neurons that derive from the same NB are rerouted within M0 layer and project to distinct columns (Fig. 1e, h–j). We hypothesize that the sister neurons express the same or similar Dscam1 isoforms, causing repulsion between neurons of the same lineage. To test this possibility, we compared projection patterns of neurites of the radial units in control and *Dscam1* null mutant clones (*dscam*^*20*^; Fig. 5a–d).

In control clones, neurites of a radial unit were defasciculated and projected to remote columns (distance = 50 μm, *n* = 8; Fig. 5a, c). Note that we measured the largest distance among multiple neurons, which is greater than the average distance between two neurons (Fig. 1l). In contrast, *Dscam1* mutant neurites were bundled in M0 layer and projected to the same or nearby columns (distance = 20 μm, *n* = 10; Fig. 5b, c), suggesting that Dscam1 is responsible for lineage-dependent repulsion.

To determine whether lineage-dependent repulsion is essential for column formation, we examined changes in columnar structure, as visualized with Ncad antibody at 48h APF, in the presence of control and *Dscam1* null mutant clones (Fig. 5d, e). We classified column morphology into normal, unclear, irregular and fused (Fig. 5f; “Methods”), and quantified column morphology (Fig. 5g). Abnormal, unclear, irregular, and fused columns were significantly increased in the presence of mutant clones.

The shape of individual columns and column arrangement were widely affected when the medulla contained *Dscam1* mutant radial units (Fig. 5e). The nonautonomous columnar defects caused by *Dscam1* mutant clones suggest that axons of the same lineage need to project to a wide range of columns under the control of Dscam1-dependent repulsion. Thus, Dscam1 is essential for lineage-dependent repulsion and subsequent column formation.

Although the neurites of a radial unit repel each other by projecting to remote columns early in the larval stage (Fig. 1e, h–j), dispersion of their cell bodies occurs between 12 and 24 h APF^11^. To examine whether the dispersion occurs in *Dscam1* mutant radial units, we compared distributions of cell bodies in control and *Dscam1* mutant clones (*dscam*^*20*^; Fig. 5h–k). In control clones, the cell bodies of a radial unit were widely distributed throughout the medulla cortex showing tangential dispersion (*n* = 3, 20 neurons/brain, SD = 42 µm; Fig. 5h, i). However, when the radial unit lacked Dscam1 function, the cell bodies remained close to each other, forming a cluster at 24 h APF (*n* = 3, 20 neurons/brain, SD = 24 µm; Fig. 5j, k). Thus, the dispersion of cell bodies also depends on Dscam1.

### Loss of Dscam1 diversity leads to columnar defects

**Fig. 6:**
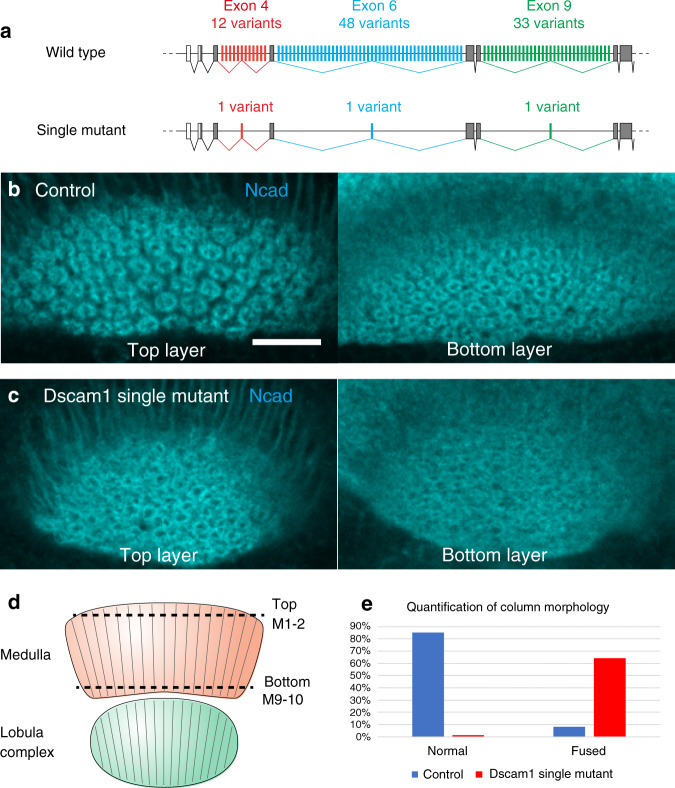
Loss of Dscam1 isoform diversity leads to defects in column formation. **a** Schematic representation of *Dscam1* gene in wild type and *Dscam1* single-isoform mutant backgrounds. Column morphology of 48 h APF pupal medulla visualized by Ncad (blue) in the M1–2 (top) and M9–10 (bottom) layers in control (**b**) and *Dscam1* single mutant backgrounds (**c**; *Dscam1*^*23*^*/Dscam1*^*3.31.8*^). Scale bars indicate 20 μm. Experiment was independently repeated at least three times with similar results (**b**, **c**). **d** Schematic representation of the optic lobe at 48 h APF. (**e**) Quantification of column morphology in the top layer. Control: 74 columns from 5 brains. Single-isoform mutant: 143 columns from 5 brains (Fisher exact text, normal *P* = 2.11 × 10^−40^, fused *P* = 5.4 × 10^−17^, irregular *P* = 0.33, unclear *P* = 0.21). Source data are provided as a Source Data file.

Previous studies have demonstrated that diversity of Dscam1 isoforms is critical for neuronal wiring using *Dscam1* mutant alleles in which the number of splice isoforms is reduced (Fig. 6a)^32^. We asked whether Dscam1 diversity is also crucial for column formation.

By combining the single-isoform mutants *Dscam*^*3.31.8*^ and a null allele, *Dscam*^*23*^, we generated a mutant background that produces only one Dscam1 isoform (Fig. 6a). We compared column shape and column arrangement as visualized with Ncad antibody at 48 h APF (Fig. 6b, c). In control brains, regular arrangement of the donut-like columns was observed (Fig. 6b, d)^7^. In contrast, shape and arrangement of the columns were significantly disorganized in the Dscam1 single-isoform backgrounds (Fig. 6c). The defects in the top layer were quantified and statistically tested (Fig. 6e). Since the bottom layer was too disorganized and column shape was unidentifiable in the mutant, quantification of column morphology was not applicable in the bottom layer (*n* = 5).

## Discussion

**Fig. 7:**
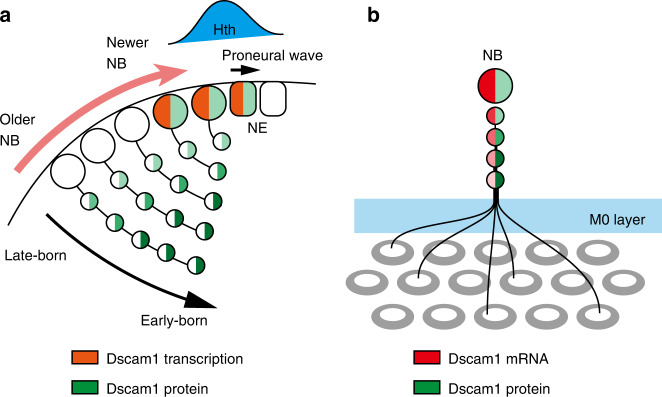
Inheritance of *Dscam1* mRNA and Dscam1 protein, and lineage depend repulsion. Schematics of *Dscam1* transcription in NEs and newer NBs under the control of Hth behind the proneural wave (**a**). Graded distribution of *Dscam1* mRNA and Dscam1 protein in neurons of the same lineage that controls lineage-dependent repulsion within M0 layer and innervation to different columns (**b**).

In this paper, we demonstrate that *dscam1* is temporally transcribed in NEs and newborn NBs under the control of the temporal transcription factor, Hth. *dscam1* mRNA and Dscam1 protein are then inherited by neurons in a lineage-dependent manner (Fig. 7). Among 20,000 splice isoforms of Dscam1, small number of isoforms are stochastically selected. Since Dscam1 is temporally expressed in NBs, neurons of the same lineage tend to share the same or similar splice isoforms, which causes homophilic binding and subsequent repulsion. As a result, neurons of the same lineage repel each other and contribute to different medulla columns (Fig. 7b).

However, our results cannot explain the following issues. First, sister neurons occasionally project to very distant regions of the medulla. For example, the sister neurons can project to the ventral and dorsal halves of the medulla neuropil at the same time (Fig. 1i). Since repulsive action of Dscam1 is triggered by its direct homophilic interaction, the projections of sister neurons over such a long distance cannot be explained solely by Dscam1 function. As-yet-unknown chemorepulsive molecules or other guidance molecules that are expressed in distinct subdomains of the medulla might work together with Dscam1. For example, Slit and Netrin act as repulsive guidance molecules during larval optic lobe development^33,34^. Optix is expressed in the dorsal and ventral subdomains of the medulla^19,35^ and may regulate the region specific expression of downstream guidance molecules.

Second, in the presence of the same Dscam1 isoform in a radial unit, the neurites are closely bundled within the medulla cortex prior to defasciculation (Fig. 1). A previous study showed that low expression levels of Dscam1 produce adhesive signal, while high expression levels provide repulsive signal^23^. Thus, the function of Dscam1 may be switched from adhesive to repulsive depending on its expression level, as demonstrated in Netrin signaling^34,36^. Consistent with this idea, Dscam1 is more strongly localized in M0 than its localization within the medulla cortex (Fig. 3c). Alternatively, an as-yet-unknown mechanism may be involved.

The waves of differentiation are observed during development of a wide variety of visual systems in animals from flies to mammals^9^. In addition, the radial unit, a group of neurons generated by a common neural stem cell, is also found during column formation in the mammalian cerebral cortex^4^, and neurons of the same lineage are dispersed later in development, as found in the fly medulla^5,6^. Thus, the mechanism demonstrated in this study combining the wave of differentiation and the temporal expression of guidance molecule encoding column identification code might be an evolutionarily conserved strategy of column formation from fly to mammalian brains.

In this study, we propose that splice diversity of Dscam1 regulates column formation through lineage-dependent repulsion. Similar but distinct mechanisms are found in the mammalian adaptive immune systems and olfactory systems. The immunoglobulin and T cell receptor (TCR) genes contain multiple gene segments that are stochastically selected and rearranged to generate variable molecules that recognize various antigens^37^. In contrast to the irreversible and permanent recombination processes found in the adaptive immune systems, the alternative splicing of Dscam1 is more flexible and reversible.

In the mouse olfactory system, each olfactory sensory neuron expresses one olfactory receptor (OR) gene out of ~1000 OR genes^38^. A single OR gene is stochastically selected by a *cis*-acting regulatory element that controls multiple genes located at a genetic locus. Furthermore, a negative feedback mechanism inactivates expression of the other OR genes after one OR gene is selected. Thus, the expression of OR genes is rigorously regulated.

Thus far, we only suggest that a single Dscam1 isoform is selected due to occupation of a single splice variant by spliceosome and temporally restricted transcription. If there is no specific mechanism that represses the expression of the other unselected isoforms, the process of lineage-dependent repulsion may not be very strictly controlled. Nevertheless, the mechanism that we propose in this study is very simple. We only need to assume the existence of multiple splice isoforms and temporally restricted transcription in stem cell-like progenitor cells. It will be interesting to determine whether similar mechanisms exist in other biological systems including column formation in mammalian brains.

## Methods

### Fly strains

Fly strains were maintained on standard *Drosophila* medium at 25 °C. The following mutant and transgenic flies were used: *dscam*^*20*^^39^, *dscam*^*21*^, *dscam*^*23*^^40^, *dscam*^*3.31.8*^, *dscam*^*10.27.25*^^32^, *hth*^*p2*^^30^, *UAS-hth*^*1-12*^^41^, *elav-Gal4*, *hs-flp, FRT42D, FRT82B, ubiGFP, tub-Gal80, UASCD8GFP*^42^, and *Ay-Gal4*^43^.

### Clonal analysis

Neurons of the same lineage were visualized by crossing *hs-flp elav-Gal4 UAS-CD8GFP; tub-Gal80 FRT42D* with *FRT42D*, *dscam*^*20*^*FRT42D*, *dscam*^*21*^*FRT42D*, *dscam*^*23*^*FRT42D* and *dscam*^*3.31.8*^*FRT42D*, and applying 34 °C 30 min heat shock (Figs. 1d, e, n, o, 5, and Supplementary Fig. 2a–e). Small number of neurons of the same lineage were visualized by crossing *hs-flp; UAS-CD2RFP UAS-GFP-Mir FRT40A* with *UAS-CD8GFP UAS-CD2-Mir FRT40A; drf-Gal4*, and applying 34 °C 30 min heat shock (Fig. 1h–k). *Dscam1* null mutant clones were generated by crossing *hs-flp; ubi-GFP FRT42D* with *dscam*^*20*^*FRT42D*, and applying 37 °C 60 min heat shock (Fig. 3, Supplementary Figs. 1b and 3). *hth* mutant clones were generated by crossing *hs-flp; ubiGFP FRT82B* flies with *hth*^*P2*^*FRT82B*, and applying 37 °C 60 min heat shock (Fig. 4b, c). *hth* overexpression clones were generated by crossing *hs-flp*; *Ay-Gal*4 *UAS-GFP* strain with *UAS-hth*^*1-12*^, and applying 37 °C 60 min heat shock (Fig. 4d, e and Supplementary Fig. 4).

### In situ RT-PCR

In situ RT-PCR was performed as described below. Larval brains were dissected in fresh PBS and promptly transferred to ice cold 4%formaldehyde/PBS solution. The brains were transferred to ice cold 4% formaldehyde/PBS solution in a tube and fixed at 4 °C overnight. The formaldehyde solution was removed, and the brains were washed with ~800 μl of –20 °C methanol and fixed in methanol at −20 °C overnight.

The brains were washed with ~800 μl of 100% ethanol twice and incubated in ~800 μl of 50% xylene/ethanol solution at room temperature for 30 min. The brains were washed with ~800 μl of 100% ethanol twice. 100% ethanol was gradually replaced with a series of 75, 50, and 25% ethanol/H_2_O solutions and H_2_O. The tube was cooled down on ice. H_2_O was replaced with ~800 μl of –20 °C 80% acetone/H_2_O solution. The tube was incubated for 10 min on ice. The acetone solution was replaced with H_2_O and the brains were washed with ~800 μl of PTw (0.1% Tween20 in PBS) twice. PTw was replaced with 4% formaldehyde/PBS solution and the brains were fixed at room temperature for 30 min. The brains were washed with ~800 μl of PTw three times and transferred to a PCR tube.

Prior to reverse transcription, the brains were incubated at 65 °C for 5 min in a solution containing 0.34 mM 3′ reverse transcription primer (see below) and 1mM dNTP mixture in H_2_O (15 μl scale), and were cooled down on ice. Adding 6 μl of 5× PrimeScript Buffer, 32U RNase inhibitor, 200U PrimeScript Reverse Transcriptase (TaKaRa) and H_2_O to the solution (30ul scale), reverse transcription was performed by incubating at 30 °C for 10 min, at 42 °C for 30–60 min and 70 °C for 15 min. The tube was cooled down on ice and the brains were washed in H_2_O.

PCR was performed in 50 μl of PCR solution containing 1× KOD Buffer, 0.2mM dNTP mixture, 2.5U KOD DNA polymerase (Thermo Fisher), 1 μM 5′ primer, 1 μM 3′ primer (see below) and 0.02 mM digoxigenin-11-dUTP (Sigma-Aldrich). Initial denaturation for 1 min at 95 °C, 20 cycles of 10 s denaturation at 98 °C, 5 s annealing at 55 °C and 50 s elongation at 68 °C, and final extension for 5min at 72 °C.

The brains were washed in PBT (0.3% TritonX in PBS) and blocked in 5–10% normal serum/PBT solution at room temperature for 30–60 min. Primary antibody reaction was performed in a solution containing mouse anti-Dig antibody (1:200), other primary antibodies and 1% normal serum in PBT at 4 °C overnight. The brains were washed in PBT four times. Secondary antibody reaction was performed in a solution containing anti-mouse FITC secondary antibody (1:200), other secondary antibodies and 1% normal serum in PBT at 4 °C overnight. The brains were washed in PBT three times. PBT was replaced with PBS, and the brains were mounted in VECTASHIELD.

### Primers

Reverse transcription of *Dscam1* was performed by using the reverse transcription primer RT11 (GTGTTGGACCTTGACGTCTT). Control in situ PCR was performed by using the exons 8–10 primers Ctrl8_5 (GCTGATTATCGAGAATGTGGAA) and Ctrl10_3 (TTCTTCCATGTAACTTGGGGTTT).

In situ PCR of *Dscam1* exon 9 was performed by using the primers Ctrl11_3 (AACTCCGGTGGAAAGGATCT) and Ds9.1 (CCTTTGATTTCGGTGAGGAA), Ds9.2 (ACGAGTTGGACATGG), Ds9.3 (ACGAGCTGGATATGGTCTCG), Ds9.4 (ACATGGTGTCCGCCTATTGT), Ds9.5 (GCGATGTCCCAATTACCAT), Ds9.6 (TCGGCAGCGAAGTCTTTAAT), Ds9.7 (GCGGAGAAGTGGCTAGTGTC), and Ds9.8 (ATCCAAGCGTTTGACTTTGG).

In situ PCR of *Dscam1* pre-mRNA was performed by the intron primers Int1_5 (ACCGCATCAGAAAACCAATC) and Int1_3 (GTGCTGTGTGTGGATTTTGC), or Int2_5 (TCATGCTCCAACACCGAATA) and Int2_3 (CAGGGCGAATTGTTTACGTT).

In situ PCR of exon 4 was performed by using the primers Exon5R (CTCTCCAGAGGGCAATACCA) and Ds4.1 (GAGGCGGATGTTAACAAGGA), Ds4.2 (ACACAAGGCATTTGTCATCC), Ds4.3 (CCTATGTAATACGCGGCAATG), and Ds4.4 (AATCGGAGGTCAACAACGAG).

In situ PCR of exon 6 was performed by using the primers Exon7R (TCCTCGACTACTGCGTCCTT) and Ds6.4 (TACGCTCCTTTGTCCAGCTC), Ds6.8 (GCAGATCCAGAGCGGAACTA), Ds6.12 (TCGAACAATGGAGGTGTCTG), Ds6.16 (TTTCTCCATGCAATGTCCTG), Ds6.20 (TGCAGGATAAGTTTGGTGTGA), Ds6.24 (AAAGGACGGTTTCAGTCACG), Ds6.28 (AGGAAGTGGGACCCTGCTAT), Ds6.32 (TCCACCGCAATACTTTGTCC), Ds6.36 (CATCGAGGTGCAAAAGTCAA), and Ds6.40 (GTCGATTAAGGCCAGCTTTG).

Reverse transcription of *Ncad* was performed by using the reverse transcription primer RT4R (GAATTGGGTCCATTGCTGTT). In situ PCR for *Ncad* was performed by using the primers Ncad_E2 (GTATCGAAGGCAATCCCACA) and Ncad_E3 (TTTGGAAATGTGCCATCCTT).

### Histochemistry

Immunohistochemistry was performed as described below. Larval brains were dissected in PBS, and fixed in 4% formaldehyde/PBT solution at room temperature for 30–60 min. The brains were washed in PBT and blocked in 5–10% normal serum/PBT solution at room temperature for 30 min. Primary antibody reaction was performed in a solution containing primary antibodies and 1% normal serum in PBT at 4 °C overnight. The brains were washed in PBT. Secondary antibody reaction was performed in a solution containing secondary antibodies (1:200) and 1% normal serum in PBT at 4 °C overnight. The brains were washed in PBT and mounted in VECTASHIELD.

Primary antibodies: rabbit anti-Hth (1:1000; Adi Salzberg, Israel Institute of Technology, Israel), rat anti-Dpn (1:100; 11D1CH11, abcam), guinea pig anti-Lsc (1:1200), rat anti-Ncad (1:20; DSHB), mouse anti-Dscam1 (1:200; S. Lawrence Zipursky, UCLA, USA), mouse anti-Dig (1:200; 21H8, abcam) and rabbit anti-GFP Alexa488 conjugated (1:1000; Invitrogen A21311) antibodies. Secondary antibodies: anti-mouse Cy3, anti-mouse FITC, anti-mouse Cy5, anti-guinea pig Cy5, anti-guinea pig FITC, anti-rat Cy5, anti-chicken Cy3 (Jackson ImmunoResearch Laboratories) antibodies.

Confocal images were obtained by Zeiss LSM880 and processed using ZEN 2.3, ImageJ 1.52a and Adobe Photoshop CC 2019.

### Image processing

Distance between pairs of neurons were measured by focusing on axons on the surface of the medulla layer visualized by Ncad staining using Straight Line and Measure tools of ImageJ according to the scale bar provided by ZEN (Fig. 1l). When the distance was >10 μm, Segmented Line tool was used to measure the distance along the surface of the medulla layer.

Signal intensity was quantified within the indicated rectangle areas by ImageJ (Figs. 2d–k and 3a, c). In Supplementary Fig. 1l–n, a circle that encompass the Dpn-positive medulla NB area was drawn using Segmented Line tool starting from the posterior end of the brain in a counter clockwise manner. Signal intensity of mRNA was measured along the circle. Removing background signals by subtracting 100, the number of mRNA expressing domain and the relative size of each expression domain were quantified for each brain sample. 100% indicates that mRNA is expressed in all NBs on the surface of the brain as found in control in situ RT-PCR (see Supplementary Fig. 1a).

Column morphology was classified into normal and abnormal columns (Fig. 5f). Normal columns show regular donut-like morphology. Abnormal columns were further clasified into unclear, irregular, and fused columns (Fig. 5f). Unclear columns do not show clear shape, while donut-like shape is distorted in irregular columns. When adjacent columns are connected, these columns are regarded as fused columns. According to this classification, column morphology was quantified in brains containing control and *Dscam1* mutant clones (Fig. 5g).

Spatial distribution of neuronal cell bodies was quantified by ImageJ as follows (Fig. 5h–k). Subtract Background and Threshold to reduce background noises below the threshold level. Fill holes, Convert to Mask and Watershed to separate neurons that are close to each other. Analyze Particles (size = 20-Infinity) to remove garbage and extract positions of cells in the X–Y coordinates^44^.

### Statistics and reproducibility

For quantification and statistical analysis, distinct brain samples were measured and analyzed as indicated in the text. Image intensities were not artificially processed except as otherwise noted. When statistics were not applicable, experiments were independently repeated at least three times with similar results.

### Reporting summary

Further information on research design is available in the Nature Research Reporting Summary linked to this article.

## Supplementary information

Supplementary Information

Peer Review File

Reporting Summary

## Data Availability

The authors declare that the data supporting the findings of this study are available within the paper, or available upon request. Source data are provided as a Source Data file. Source data are provided with this paper.

## Source data

Source Data
