# Supplementary material for: Dscam1 establishes the columnar units through lineage-dependent repulsion between sister neurons in the fly brain
Source: Nat Commun. 2020 Aug 13;11:4067. doi: 10.1038/s41467-020-17931-w (PMC7426427; doi:10.1038/s41467-020-17931-w)
Supplement: Supplementary file 1 — Supplementary Information [file 41467_2020_17931_MOESM1_ESM.pdf]

Supplementary Information

Dscam1 establishes the columnar units through lineage-dependent repulsion between sister neurons in the fly brain

Liu et al.

Supplementary Information

Supplementary Figures

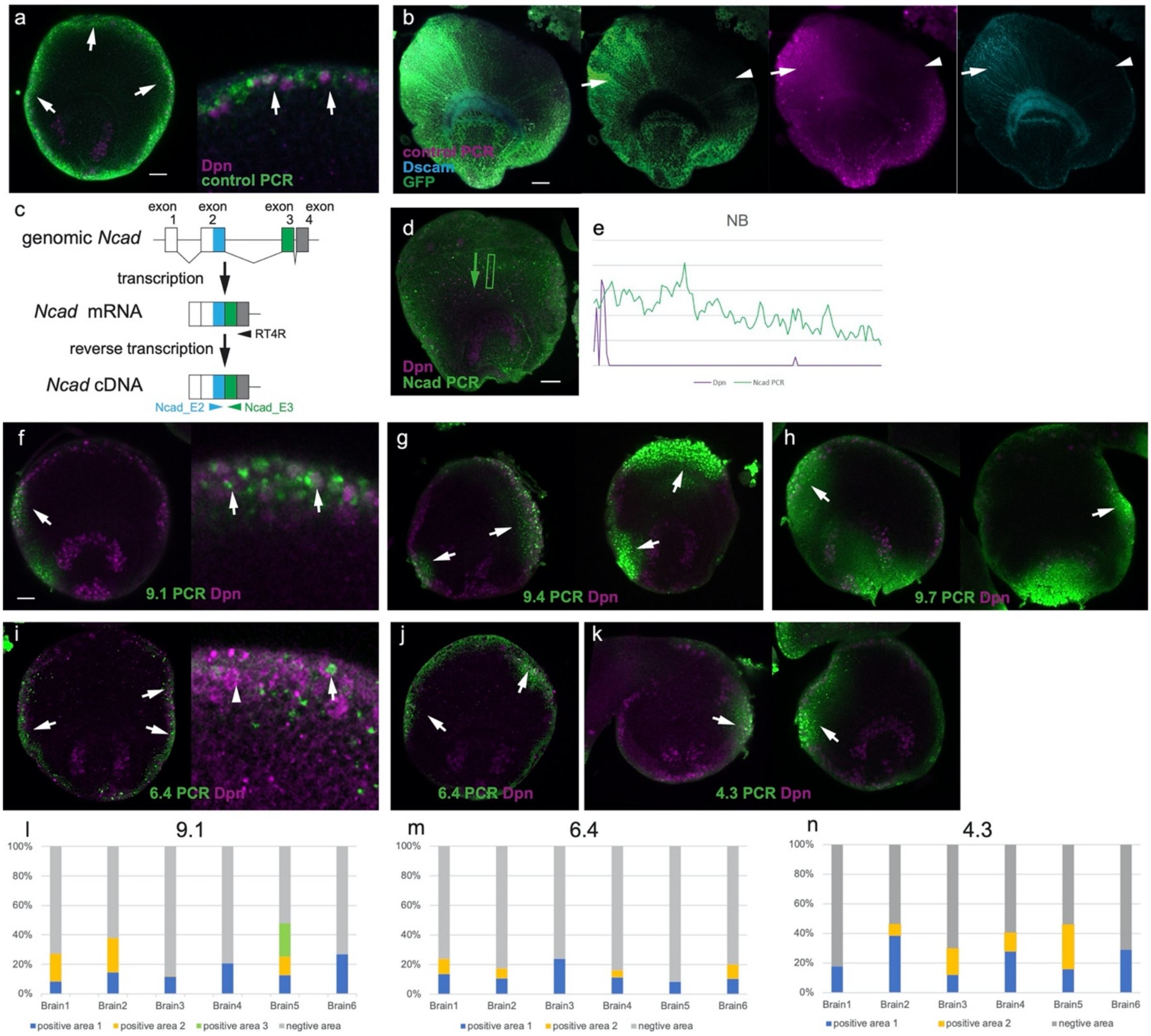

Supplementary Figure 1. ***in situ*** **RT-PCR for** ***Dscam1*** **and** ***Ncad.***

(a) *Dscam1* control PCR (green), Dpn (magenta). Dotted mRNA signals inside NBs are magnified (arrows). (b) Signals for *Dscam1* control PCR (magenta) and Dscam1 protein (blue) are lost in *Dscam1* mutant clones indicated by the loss of GFP signal (green). Control and mutant cells are indicated by arrows and arrowheads, respectively. (c) Schematic of *Ncad* gene structure and primers design for *in situ* RT-PCR. (d) *Ncad in situ* RT-PCR (green), Dpn (blue). (e) Quantification of signal intensity in the box in (d). Background signal was subtracted for Dpn. (f-n) *in situ* RT-PCR signals for various *Dscam1* exon variants are shown in green (arrows). NBs are visualized by Dpn in magenta. (f) Dotted mRNA signal of exon 9.1 in NBs. (g, h) mRNA signals of exons 9.4 and 9.7 are found in a part of medulla NBs. (i) Dotted mRNA signal of exon 6.4 in NBs. Arrowhead indicates a mRNA negative NB within mRNA positive area. (j, k) mRNA signals of exons 6.4 and 4.3 are found in a part of medulla NBs. (l-n) Quantification of the expression domains of exons 9.1, 6.4 and 4.3. Scale bars in (a, b, d, f-k) indicates 20µm. Experiment was independently repeated at least three times with similar results (a, b, d, f-k). Source data are provided as a Source Data file (e, l, m, n).

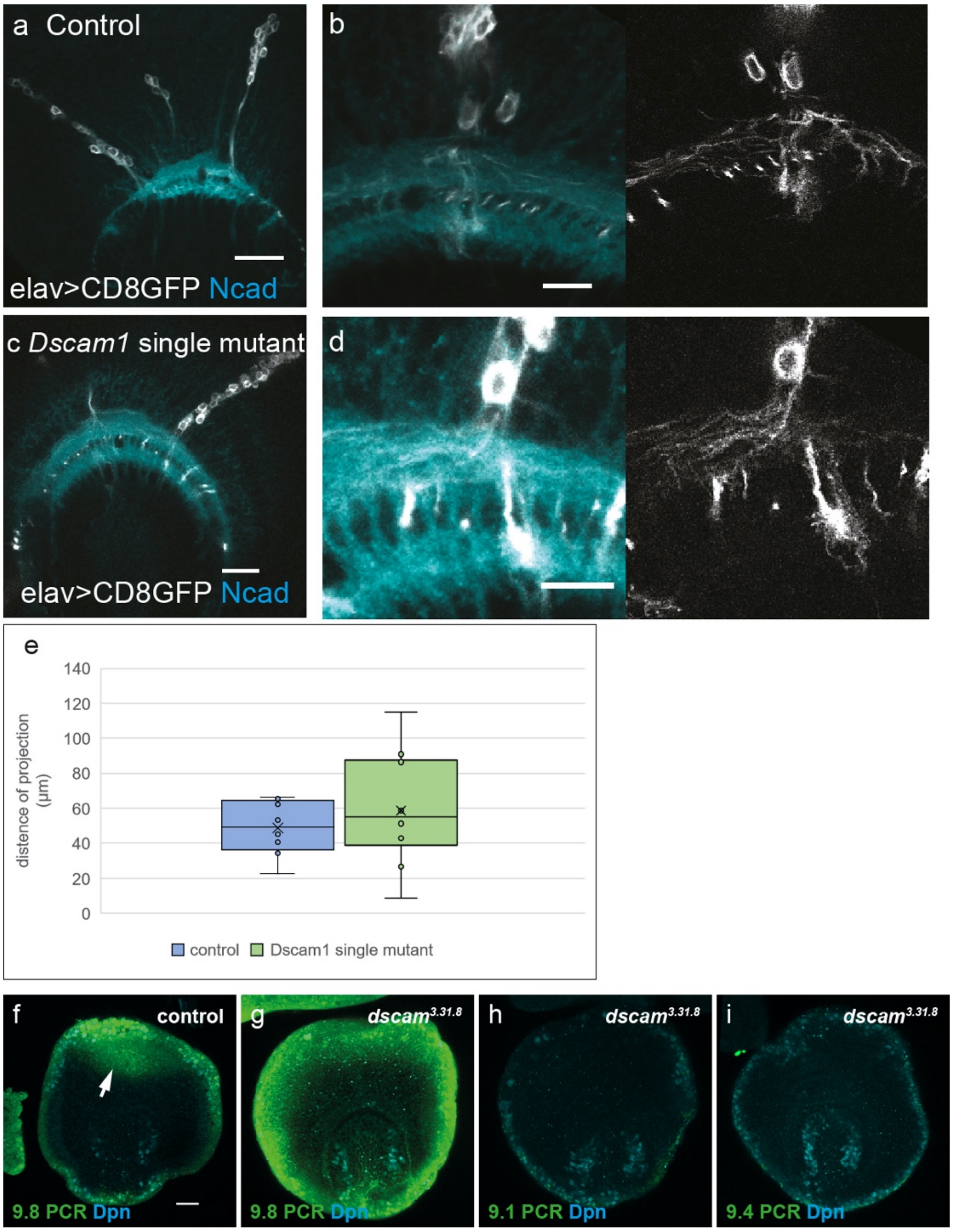

Supplementary Figure 2. Projection patterns and expression of *Dscam1* exon variants in *Dscam1* single isoform mutant.

(a-d) Neurons of the same lineage are visualized by *elav-Gal4* MARCM clones (GFP in white). Lateral views showing the neuron layer of L3 larval brain. Ncad (blue) visualizes the neuropil structure. Control, n=21 (a, b) and *Dscam1* single isoform mutant clones, n=19 (c, d). Wide spread tangential projections in the M0 layer are found in both control and mutant clones (b, d). (e) Quantification of the distance between neurites of the same lineage. Control: n=8, average projection distance=50µm, single isoform mutant: n=10, average projection distance=58µm (t-test, two-sided, P=0.40). Center line, median; box limits, upper and lower quartiles; whiskers, maxium and minimum. Source data are provided as a Source Data file. (f-i) mRNA signals of exon 9 variants (green) and Dpn (blue) in control (f) and *Dscam1* single isoform background. (f) exon 9.8 in control (n=10). (g-i) exons 9.8, 9.1, 9.4 in $Dscam1^{3.31.8}$/ $Dscam1^{20}$ background (n=10, 10, 8), respectively. Scale bars indicate 20µm.

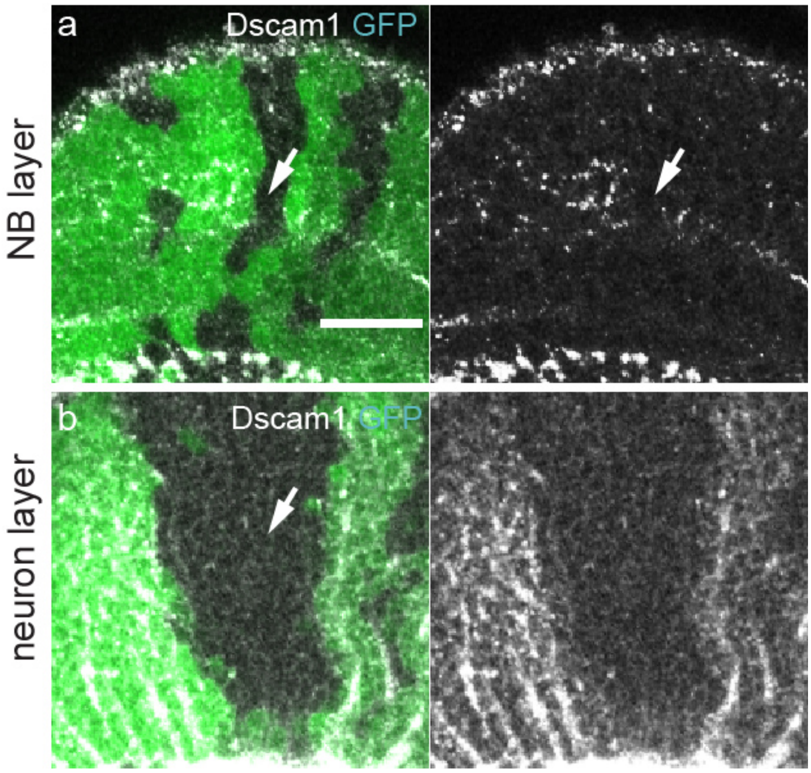

Supplementary Figure 3. Dscam1 null mutant clones.

(a, b) *Dscam1* null mutant clones visualized by the absence of GFP (green; arrows) in lateral views of L3 larval brains. Dscam1 signal (white) is eliminated in mutant clones in the NB layer (a; arrows). Residual background Dscam1 signal remains in mutant clones in the neuron layer (b; arrows). Scale bars indicate 20µm. Experiment was independently repeated at least three times with similar results.

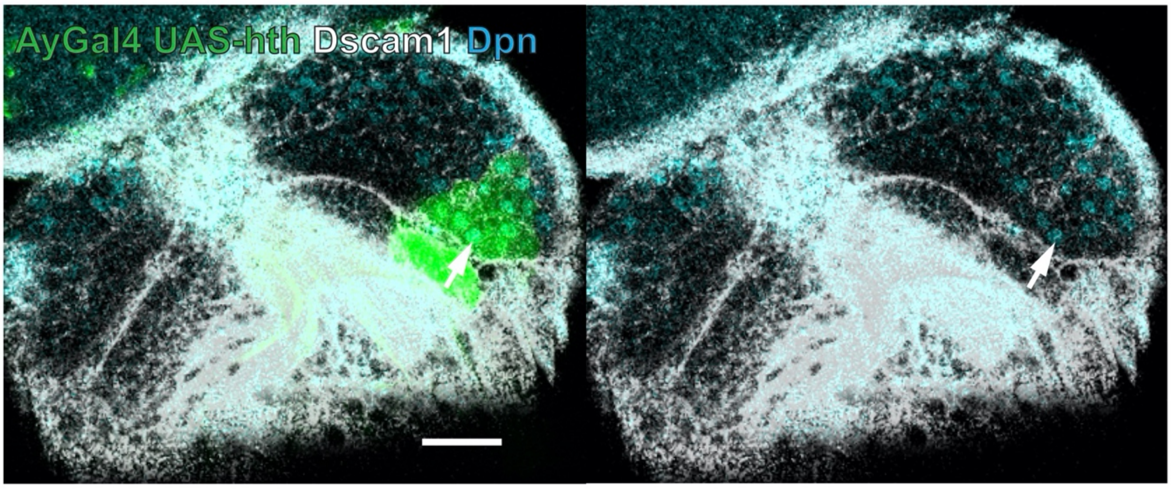

Supplementary Figure 4. Ectopic expression of *hth* causes Dscam1 upregulation and premature NB differentiation.

A *hth* expressing clone visualized by GFP (green) in a lateral view of L3 larval brain in the NB layer induced ectopic Dscam1 upregulation (white) and premature NB differentiation (Dpn; blue; arrows). Scale bars indicate 20μm. Experiment was independently repeated at least three times with similar results.
