# Supplementary material for: Dscam1 establishes the columnar units through lineage-dependent repulsion between sister neurons in the fly brain
Source: Nat Commun. 2020 Aug 13;11:4067. doi: 10.1038/s41467-020-17931-w (PMC7426427; doi:10.1038/s41467-020-17931-w)
Supplement: Supplementary file 2 — Peer Review File [file 41467_2020_17931_MOESM2_ESM.docx]

Peer Review Information

**Journal:** Nature Communications

**Manuscript title:** Dscam1 establishes the columnar units through lineage-dependent repulsion between sister neurons in the fly brain

**Corresponding author name(s):** Prof Makoto Sato

**Editorial notes:**

**Transferred manuscripts**

This manuscript has been previously reviewed at another journal that is not operating a transparent peer review scheme. This document only contains reviewer comments and rebuttal letters for versions considered at *Nature Communications*.

**Reviewer comments & decisions:**

**Reviewer comments, first version:**

Reviewers' comments:

Reviewer #1 (Remarks to the Author):

Liu et al. show that the axons of clonally related neurons in the medulla tend to defasciculate and project to distant regions. They use an in situ RT-PCR method to show that the homophilic repulsion molecule Dscam1 is primarily transcribed in newly born neuroblasts, with individual splice isoforms expressed in groups of adjacent neuroblasts. Dscam1 protein is present in neurons and localized to their axons. The authors report that Homothorax, the first transcription factor expressed in newly born neuroblasts, is necessary for Dscam1 expression and overexpressing Hth leads to upregulation of Dscam1, although this may be a consequence of premature neuronal differentiation. In Dscam1 mutant clones, projections from related neurons are less widely dispersed than in control clones, and tangential migration of the cell bodies is also reduced. Eliminating Dscam1 isoform diversity disrupts the columnar organization of the medulla, although clones of neurons that express a single isoform still show dispersed axonal projections.
These results are potentially interesting, as they suggest that a short burst of Dscam1 transcription in neuroblasts could result in inheritance of the same splice isoforms by their daughter neurons, promoting the dispersal of their neurites. However, there is a general lack of quantification in the paper. The distance between neurites of neurons in the same radial unit (Fig. 1h-k) should be quantified. The numbers reported for the hth clones show that most hth mutant clones do not lose Dscam1 expression, and most Hth overexpressing clones do not upregulate Dscam1 expression; this does not provide strong support for the authors’ conclusion that Hth regulates Dscam1. Positively marked hth mutant clones would more convincingly identify the mutant neurites. Figure 5a-d also need quantification of the distance between neurites and the organization of columns in Dscam1 mutant clones.
Because the in situ RT-PCR technique is new, controls should be shown to make sure it is working as expected. Are the control and intron signals lost in Dscam1 mutant clones? Is the exon 9.1 signal lost in the single isoform mutant background? Examples of other single exon PCRs should be shown. Do the authors have an explanation for why a cluster of adjacent neuroblasts would splice in the same exon 9 variant?


Reviewer #2 (Remarks to the Author):

The development of high level brain structure is a critical question in neural development. The paper by Liu et al. investigates the role of the Dscam1 gene in the formation of columns in the medulla of Drosophila. The authors find that Dscam1 is necessary for the defasciculation of tightly bundled axons of the same neuronal lineage in the temporary M0 layer of the medulla. This finding appears functionally analogous to sister axon branch segregation during the formation of mushroom body lobes, which also requires Dscam1 function at a distinct location. However, Dscam1 is required to ensure the axons project long distances after defasciculation and failure to do so disrupts column formation. Utilizing repulsion by homophilic cell adhesion molecules may be a general principle in column formation.

The paper is well-written and beautifully photographed but lacks some of the necessary quantification to support their conclusions. The authors employ a potentially game-changing technical approach in the form of in situ RT-PCR, but additional data should be shown. The prior literature is adequately cited, including a paper from an independent group that predicted the role of Dscam in the medulla. Overall, the data supports the conclusions drawn and is suitable for publishing in Nature Communications.

Data Quantification. For the labeled clones shown in figure 1, quantification of the data by Sholl analysis or a similar approach should be undertaken to support the conclusions stated in the text, e.g. “Usually, the sister neurons… do not form projections to the same columns”, “neurons of the same lineage often project to”.
For figure 4, can the upregulation of Dscam be quantified relative to the surrounding tissue? Why is Dscam upregulation in Fig,.4D and E limited to a small area of the GFP+ clone?
Fig. 6 - how many brains were examined? Can the disruption to the columns be quantified in some way, such as number of Ncad positive domains that inappropriately make substantial contact with neighboring domains?

In situ RT-PCR is an approach that appears to have been largely limited to plants and fungal material. I cannot find an example where it has previously been used in Drosophila. For alternatively spliced genes like Dscam, the opportunity to visualize expression of different isoforms is very exciting. The approach should also be widely accessible, unlike the deep sequencing approaches that have spatial resolution, which are limited a few labs. It is therefore important that this part of the paper is adequately documented. How many samples were examined for each primer pair? Is there a way to quantify the data, even if only to confirm that each sample displayed Dscam1 signal restricted to the neuroblast domain? The pattern for the variable exon specific primer (Fig. 2h) is intriguing, showing two large regions of neurons expressing the same isoform. Based on sequencing results, I imagine many researchers would have predicted a salt and pepper expression pattern of different isoforms. There may be significant biological implications if isoform usage is set at the earliest stages of neuroblast development. The authors mention that they tested eight splice variants for exon 9; this data should be quantified, such as number and approximate size of region expressing the specific variant. The images should be included in the supplemental data.


Minor comments
Near the end of the introduction, Dscam is described as a gene putatively responsible for Down syndrome. There is no citation for this statement, and it might be better phrased as “significantly contributing to some of the phenotypes observed in Down syndrome” as other genes appear to have stronger overall effects.

The introduction wonderfully puts the work into a broader context of neuronal organization. A formal definition of the medulla column should be given in the introduction to avoid confusion for the reader between the columns of differentiating neurons and the columns in the neuropil. Are the Ncad negative regions cell bodies or dendrites? The location of columns should be labeled in figure 1. Additional terms such as the medulla cortex should also be defined for clarity.

Results: briefly explain why MARCM is used (to genetically label a small subset of cells).

Last sentence of second results paragraph – reminiscent may be a better word than luminescent.

Full gene names should be presented once; e.g. homothorax/hth.

A similar pattern of mRNA expression in neuroblasts has been observed for the frazzled axon guidance receptor in the embryo (Joly et al. 2007 Dev. Biol. 301:542).

Figure legends could generally include a little bit more detail to allow the reader to fully comprehend the figure. For example:
Fig. 2. State that in situ RT-PCR is being used.
Fig. 4. State the GAL4 driver used to make the clones.


Reviewer #3 (Remarks to the Author):

How large, partly repetitive arrays of neurons are properly wired up remains still largely unknown, even though several key pathways and principles have been identified in the past decades. One important part of the early decision on adhesion/repulsion is regulated by the adhesion pathway of the Down syndrome cell adhesion molecule (Dscam) gene.
In the current manuscript Liu and colleagues investigate the role of DSCAM in the developing optic ganglia – more specifically in the medulla- of the fruit fly. First, they provide evidence that neurons from the same lineage project into distinct columns, suggesting “lineage-dependent repulsion”. They provide evidence that DSCAM is involved in the process as clones expressing the same isoform show disorganized projections. In an array of experiments, they build up a model in which the DSCAM is expressed in the NB, but the protein is required subsequently in the neuron; along the same lines they suggest that within a NB lineage, similar isoforms are expressed.

The manuscript is intriguing and provides insight into the mechanisms that coordinate the structured development of the optic lobes. However, the core messages are not without problems, particularly since the claims of how systems appear to work are not quite intuitive. My main concern lies in the dynamic of RNA/protein expression.
While the DSCAM and Hth clones indeed show that the protein detection works, the RNA data is not as clear. Similarly, it is unclear how protein can be synthesized without RNA- at least at some stage of immature neurons there must be RNA present.
I feel it would be important to further substantiate the claim/model, in particular molecular and/or genetic evidence.

- It is intrinsically hard to have a NB specific experiment, and have the progeny express the protein. In the case of DSCAM it However, if indeed the RNA is only required in NBs one line of evidence could be gained by a neuron-specific manipulation. While this would be a negative result, showing that DSCAM expression is dispensable from neurons (what would be expected from the model) would support the notion.

- While the approach of using in situ PCR would be intrinsically elegant to distinguish different DSCAM isoforms the data displayed is not particularly clear. For instance, it is unclear if the approach allows to detect RNAs in deeper layers as overall the signal seems to be strong at the surface cell layer (maybe layer 2). Thus, the indirect quantification that is displayed may be faulty.
Since much of the argumentation of the manuscript is based on the temporal dynamics of DSCAM RNA and protein a clarification of this point would be critical. For instance, control experiments with other markers (e.g. DPN, Lsc and more general ELAV) would support the notion of the authors.

- The experiment shown in figure 2h to test exon 9.1 RNA detection is not convincing. There appears to be signal on several NBs on one side of the brain. If so, this appears to be a domain-like expression and not in agreement with sparse stochasticity.

- The layout of figures and text are not well adherent; particularly the initial sections jump between figures and panels. A more logical layout would be more appealing and making it easier for a reader to follow the manuscript.

- Subsequent clonal analysis is clearer.

- The beginning of the abstract jumps directly to the columnar organization without any introductory or explanatory sentence. It would be beneficial to provide some context.

**Author rebuttal, first version:**

Reviewer #1 (Remarks to the Author):
Liu et al. show that the axons of clonally related neurons in the medulla tend to defasciculate and project to distant regions. They use an in situ RT-PCR method to show that the homophilic repulsion molecule Dscam1 is primarily transcribed in newly born neuroblasts, with individual splice isoforms expressed in groups of adjacent neuroblasts. Dscam1 protein is present in neurons and localized to their axons. The authors report that Homothorax, the first transcription factor expressed in newly born neuroblasts, is necessary for Dscam1 expression and overexpressing Hth leads to upregulation of Dscam1, although this may be a consequence of premature neuronal differentiation. In Dscam1 mutant clones, projections from related neurons are less widely dispersed than in control clones, and tangential migration of the cell bodies is also reduced. Eliminating Dscam1 isoform diversity disrupts the columnar organization of the medulla, although clones of neurons that express a single isoform still show dispersed axonal projections. 
These results are potentially interesting, as they suggest that a short burst of Dscam1 transcription in neuroblasts could result in inheritance of the same splice isoforms by their daughter neurons, promoting the dispersal of their neurites. However, there is a general lack of quantification in the paper. The distance between neurites of neurons in the same radial unit (Fig. 1h-k) should be quantified.

We directly quantified the distance between axons that derive from the same radial unit as quantified and summarized in Fig. 1l, m, and described in line 141-154.

We quantified the distance between sister neurons that derive from the same radial unit by focusing on brain samples that contain very small number of isolated clones (Fig. 1l, m). When the axons of multiple neurons were fused and indistinguishable, we regarded the distance as 0mm. When individual axons were distinguishable, the distance between axon shafts on the surface of the medulla layer was measured (Fig. 1l). Minimum distance between distinguishable axons were 2mm. According to Ncad staining, the distance between columns is about 5um in larval medulla (Fig. 1g). Among 110 pairs of neurons, 11 and 37 pairs were 0mm and 2-5mm distant, respectively. 62 pairs projected to the medula layer showing distance greater than 5mm (Fig. 1l). Note that 2-5mm distance does not necessarily mean that they are projecting to the same column, because they may still project to the adjacent domains of the neighboring columns.

The distribution of the distance between distinguishable pairs of axons is plotted in Fig. 1m (0mm distance is omitted). Median and average of the distance are 5.78 and 8.50um, respectively, and are greater than 5um, the distance between columns.

The numbers reported for the hth clones show that most hth mutant clones do not lose Dscam1 expression, and most Hth overexpressing clones do not upregulate Dscam1 expression; this does not provide strong support for the authors’ conclusion that Hth regulates Dscam1.

We consider two possibilities. First of all, the mutant alle we used is the most commonly used allele, but is hypomorphic. Secondly, there might be additional unknown factors that act redundantly with *hth*. We added the following sentences in line 317-320. At least, *hth* plays important role in regulating Dscam1 expression.

Note that the *hth* mutant used in this study (*hth^P2^*) is the most commonly used allele, but is hypomorphic (Fly Base). The incomplete loss of Dscam1 expression may be due to the hypomorphic nature of the *hth* mutant. Or, there might be additional unknown factors that act partially redundantly with *hth*.

Positively marked hth mutant clones would more convincingly identify the mutant neurites.

In this experiment, we need to focus on the cell bodies to examine the difference of Dscam1 expression in NBs and NEs (Fig. 4b-c). Therefore, a cytoplasmic GFP was used to negatively label *hth* mutant clones. Positive labeling would not make difference in the results.

Additionally, *hth* mutant MARCM clones may show defects in neurite repulsion as demonstrated in *Dscam1* MARCM clones (Fig. 5a-c). However, interpretation of the data would be difficult, because *hth* mutation dramatically changes neural identity and projection patterns of neurons (Hasegawa 2011; Hasegawa 2013).

Figure 5a-d also need quantification of the distance between neurites and the organization of columns in Dscam1 mutant clones.

We carefully considered quantification of the distance between neurites and column morphology as follows. The distance of neurite projection in Fig. 5a-b are quantified in Fig. 5c and statistically tested.

Line 342-346: In the control clones, the neurites of a radial unit were defasciculated and projected to remote columns (distance=50μm, n=8; Fig. 5a, c). In contrast, the *Dscam1* mutant neurites were bundled in the M0 layer and projected to the columns that were close to the cell bodies of the radial unit (distance=20μm, n=10; Fig. 5b, c), suggesting that Dscam1 is responsible for the lineage-dependent repulsion between the daughter neurons.

Line 692-694: (c) Quantification of the distance between neurites of the same lineage. Control: n=8, average projection distance=50mm. *Dscam1* mutant: n=10, average projection distance=18mm (t-test, P<0.05).

To rigorously quantify column morphology, we considered to focus on 48h APF brains, because the column shape becomes cleaer compared with the earlier stages. Column morphology was classified into 'normal' and 'abnormal' columns. 'abnormal' columns were further classified into 'unclear', 'irregular' and 'fused' columns (Fig. 5f). According to this classification, column morphology was quantified and statistically tested in Fig. 5g.

Line 349-357: We classified column morphology into 'normal' and 'abnormal' columns (Fig. 5f). 'normal' columns show regular donut-like morphology. In contrast, 'abnormal' columns were further clasified into 'unclear', 'irregular' and 'fused' columns (Fig. 5f). 'unclear' columns do not show clear shape, while donut-like shape is distorted in 'irregular' columns. When adjacent columns are connected showing no gap between them, these columns are regarded as 'fused' columns. According to this classification, column morphology was quantified in brains containing control and *Dscam1* mutant clones (Fig. 5g). The results demonstrate that 'abnormal', 'unclear', 'irregular', and 'fused' columns were significantly increased in the presence of mutant clones.

Line 697-700: (f) Classification of column morphology. In contrast to 'normal' columns, 'abnormal' columns are classified into 'irregular', 'fused' and 'unclear' columns. (g) Quantification of column morphology. Control: 87 columns from 3 brains. *Dscam1* mutant clones: 136 columns from 3 brains (Fisher exact test, P<0.05).

Because the in situ RT-PCR technique is new, controls should be shown to make sure it is working as expected. Are the control and intron signals lost in Dscam1 mutant clones?

Since in situ RT-PCR is technically tough, combining it with clonal analysis is very hard because mutant clones are randomly and infrequently induced. We nevetheless tried control PCR in *Dscam1* null mutant clones, and found that it is indeed abolished in mutant areas (Fig. S1b), suggesting that *in situ* RT-PCR specifically detects *Dscam1* mRNA (line 213-216).

To confirm the validity of our *in situ* RT-PCR technique, we examined *Dscam1* mRNA signal in clones homozygous for *Dscam^20^*, a null mutant of *Dscam1* (Fig. S1b). Compared with the control cells, signals for Dscam1 protein and *Dscam1* mRNA as visualized by *in situ* RT-PCR were abolished, suggesting that *in situ* RT-PCR specifically detects *Dscam1* mRNA.

Since Ncad is widely expressed in medulla neurons, we also visualized *Ncad* mRNA as a control by using *in situ* RT-PCR (Fig. S2c-e; line 217-223).

We also visualized *Ncad* mRNA by using *in situ* RT-PCR (Fig. S1c-e). Consistent with Ncad protein expression in medulla neurons, we observed strong *Ncad* mRNA signal in the inner region of the medulla cortex. Relatively uniform *Ncad* mRNA signal throughout the medulla strongly suggests that the sharp decrease of *Dscam1* mRNA signal inside the brain indeed recapitulates *Dscam1* expression (Fig. 2g-i). The gradual decrease of *Ncad* mRNA signal inside the brain might be caused by inefficient penetration of the reagents inside the brain samples.

Is the exon 9.1 signal lost in the single isoform mutant background?

This is a particularly good suggestion that reminds us to examine the validity of our *in situ* RT-PCR technique. mRNA for exon 9.1 was lost in the single isoform background. In addition to exon 9.1, we also examined exon 9.8 in the single isoform mutant background. Interestingly, exon 9.8 was uniformly expressed in the medulla NBs. Expression of various exon variants were examined in the single isoform mutant background as shown in Fig. S2f-i and line 256-265.

In wild type condition, we assume that the eighth variant of exon 9 (exon 9.8) is stochastically chosen upon alternative splicing. In contrast, the single isoform mutant, *dscam^3.31.8^*, lacks all variants of exon 9 except for 9.8. We next asked what happens to the expression pattern of exon 9.8 in this single variant mutant background (*dscam^3.31.8^/dscam^20^*). Surprisingly, mRNA for exon 9.8 was uniformly detected in the medulla NBs forming a circle encompassing the entire larval brain hemisphere (Fig. S2f, g), suggesting that exon 9.8 is always chosen in the absence of the other variants of exon 9. Consistently, mRNA for exon 9.1, 9.4, and 9.7 were not detected in the same mutant background (Fig. S2h, i and not shown). Taken together, these findings support our hypothesis that neurons of same lineage express similar Dscam1 isoforms.

Examples of other single exon PCRs should be shown.

To improve the data of single exon PCR, we not only repeated exon 9 RT-PCR, but also added results of exon 4 and exon 6. We repeated in situ RT-PCR experiments and quantified the results as added in Fig. S1f-n and line 234-237.

We repeated the same experiment for 22 splice variants from exon 4 (4 variants), 6 (10 variants) and 9 (8 variants.; Material and methods). At least ten samples were observed for each variant, and we obtained essentially the same results as quantified in Fig. S1l-n, suggesting that an alternative splice variant is stochastically chosen.

Do the authors have an explanation for why a cluster of adjacent neuroblasts would splice in the same exon 9 variant?

We consider the following possibilities about the clustered expression patterns of exon variants. Since Dscam1 transcription starts in NEs, which quickly divide symmetrically, multiple NBs may share the same exon variant. Or, there might be additional mechanisms. We added the following sentences in line 238-244.
If the stochastic choise of an alternative exon solely occurs in NBs, a salt-and-pepper-like pattern should appear. Indeed, closer look at their expression patterns occasionally reveals a lack of *in situ* RT-PCR signals in an expression domain (Fig. S1i). However, this may be due to the cell cycle dependent changes in mRNA distribution. Since Dscam1 expression is initiated in NEs (Fig. 2d-f), which divide symmetrically, a group of NBs is supposed to share the same exon variant. Or, there might be unknown mechanisms that provide a bias on the choise of alternative exons in NEs.

Reviewer #2 (Remarks to the Author):
The development of high level brain structure is a critical question in neural development. The paper by Liu et al. investigates the role of the Dscam1 gene in the formation of columns in the medulla of Drosophila. The authors find that Dscam1 is necessary for the defasciculation of tightly bundled axons of the same neuronal lineage in the temporary M0 layer of the medulla. This finding appears functionally analogous to sister axon branch segregation during the formation of mushroom body lobes, which also requires Dscam1 function at a distinct location. However, Dscam1 is required to ensure the axons project long distances after defasciculation and failure to do so disrupts column formation. Utilizing repulsion by homophilic cell adhesion molecules may be a general principle in column formation.

The paper is well-written and beautifully photographed but lacks some of the necessary quantification to support their conclusions. The authors employ a potentially game-changing technical approach in the form of in situ RT-PCR, but additional data should be shown. The prior literature is adequately cited, including a paper from an independent group that predicted the role of Dscam in the medulla. Overall, the data supports the conclusions drawn and is suitable for publishing in Nature Communications.

Data Quantification. For the labeled clones shown in figure 1, quantification of the data by Sholl analysis or a similar approach should be undertaken to support the conclusions stated in the text, e.g. “Usually, the sister neurons… do not form projections to the same columns”, “neurons of the same lineage often project to”.

We can unambiguously identify the axon terminals and quantify their distance using imageJ and ZEN by using Ncad staining as a marker. Details of the quantification method was added to Image processing section of Materials and methods (line 588-591).

Distance between pairs of neurons were measured by focusing on axons on the surface of the medulla layer visualized by Ncad staining using 'Straight Line' and 'Measure' tools of ImageJ according to the scale bar provided by ZEN (Fig. 1l). When the distance was greater than 10μm, 'Segmented Line' tool was used to measure the distance along the surface of the medulla layer.

Using this method, we directly quantified the distance between axons that derive from the same radial unit as quantified and summarized in Fig. 1l, m, and described in line 141-154.

We quantified the distance between sister neurons that derive from the same radial unit by focusing on brain samples that contain very small number of isolated clones (Fig. 1l, m). When the axons of multiple neurons were fused and indistinguishable, we regarded the distance as 0mm. When individual axons were distinguishable, the distance between axon shafts on the surface of the medulla layer was measured (Fig. 1l). Minimum distance between distinguishable axons were 2mm. According to Ncad staining, the distance between columns is about 5um in larval medulla (Fig. 1g). Among 110 pairs of neurons, 11 and 37 pairs were 0mm and 2-5mm distant, respectively. 62 pairs projected to the medula layer showing distance greater than 5mm (Fig. 1l). Note that 2-5mm distance does not necessarily mean that they are projecting to the same column, because they may still project to the adjacent domains of the neighboring columns.

The distribution of the distance between distinguishable pairs of axons is plotted in Fig. 1m (0mm distance is omitted). Median and average of the distance are 5.78 and 8.50um, respectively, and are greater than 5um, the distance between columns.

For figure 4, can the upregulation of Dscam be quantified relative to the surrounding tissue? Why is Dscam upregulation in Fig,.4D and E limited to a small area of the GFP+ clone?

Quantification of Dscam1 upregulation was added to Fig. 4f-i. There might be additional factors that upregulate Dscam1 together with Hth. The following explanation was added (line 330-333):

While Hth is widely expressed in NEs, Dscam1 expression is upregulated in a part of NEs prior to NB differentiation. Thus, Hth expression in NEs may not be sufficient to induce Dscam1 expression. The other unknown factor might be necessary to cause the full induction of Dscam1 expression.

Fig. 6 - how many brains were examined? Can the disruption to the columns be quantified in some way, such as number of Ncad positive domains that inappropriately make substantial contact with neighboring domains?

Column morphology was classified based on Ncad staining as shown in Fig. 5f. The defects were quantified and presented in Fig. 6e. The following sentences were added.

Line 389-393: The defects in the top layer was quantified and statistically tested (Fig. 6e). Since the bottom layer was too disorganized and the column shape was unidentifiable in the mutant, quantification of column morphology was not applicable in the bottom layer (n=5).

Line 712-714: (e) Quantification of column morphology in the top layer. Control: 74 columns from 5 brains. Single isoform mutant: 143 columns from 5 brains (Fisher exact text, P<0.05 for 'normal' vs 'abnormal' and 'fused' vs 'non-fused').

In situ RT-PCR is an approach that appears to have been largely limited to plants and fungal material. I cannot find an example where it has previously been used in Drosophila. For alternatively spliced genes like Dscam, the opportunity to visualize expression of different isoforms is very exciting. The approach should also be widely accessible, unlike the deep sequencing approaches that have spatial resolution, which are limited a few labs. It is therefore important that this part of the paper is adequately documented. How many samples were examined for each primer pair? Is there a way to quantify the data, even if only to confirm that each sample displayed Dscam1 signal restricted to the neuroblast domain? The pattern for the variable exon specific primer (Fig. 2h) is intriguing, showing two large regions of neurons expressing the same isoform. Based on sequencing results, I imagine many researchers would have predicted a salt and pepper expression pattern of different isoforms.

There may be significant biological implications if isoform usage is set at the earliest stages of neuroblast development. The authors mention that they tested eight splice variants for exon 9; this data should be quantified, such as number and approximate size of region expressing the specific variant. The images should be included in the supplemental data.

To improve the data of single exon PCR, we not only repeated exon 9 RT-PCR, but also added results of exon 4 and exon 6. We repeated in situ RT-PCR experiments and quantified the results as added in Fig. S1f-n and line 234-244.

We repeated the same experiment for 22 splice variants from exon 4 (4 variants), 6 (10 variants) and 9 (8 variants.; Material and methods). At least ten samples were observed for each variant, and we obtained essentially the same results as quantified in Fig. S1l-n, suggesting that an alternative splice variant is stochastically chosen.

We consider the following possibilities about the clustered expression patterns of exon variants. Since Dscam1 transcription starts in NEs, which quickly divide symmetrically, multiple NBs may share the same exon variant. Or, there might be additional mechanisms. We added the following sentences in line 238-244.

If the stochastic choise of an alternative exon solely occurs in NBs, a salt-and-pepper-like pattern should appear. Indeed, closer look at their expression patterns occasionally reveals a lack of *in situ* RT-PCR signals in an expression domain (Fig. S1i). However, this may be due to the cell cycle dependent changes in mRNA distribution. Since Dscam1 expression is initiated in NEs (Fig. 2d-f), which divide symmetrically, a group of NBs is supposed to share the same exon variant. Or, there might be unknown mechanisms that provide a bias on the choise of alternative exons in NEs.

Additionally, following experiments were added to verify the reliability of our in situ RT-PCR method. We tried control *Dscam1* RT-PCR in *Dscam1* null mutant clones, and found that it is abolished in mutant areas (Fig. S1b), suggesting that *in situ* RT-PCR specifically detects *Dscam1* mRNA (line 213-216).

To confirm the validity of our *in situ* RT-PCR technique, we examined *Dscam1* mRNA signal in clones homozygous for *Dscam^20^*, a null mutant of *Dscam1* (Fig. S1b). Compared with the control cells, signals for Dscam1 protein and *Dscam1* mRNA as visualized by *in situ* RT-PCR were abolished, suggesting that *in situ* RT-PCR specifically detects *Dscam1* mRNA.

Since Ncad is widely expressed in medulla neurons, we also visualized *Ncad* mRNA as a control by using *in situ* RT-PCR (Fig. S2c-e; line 217-223).

We also visualized *Ncad* mRNA by using *in situ* RT-PCR (Fig. S1c-e). Consistent with Ncad protein expression in medulla neurons, we observed strong *Ncad* mRNA signal in the inner region of the medulla cortex. Relatively uniform *Ncad* mRNA signal throughout the medulla strongly suggests that the sharp decrease of *Dscam1* mRNA signal inside the brain indeed recapitulates *Dscam1* expression (Fig. 2g-i). The gradual decrease of *Ncad* mRNA signal inside the brain might be caused by inefficient penetration of the reagents inside the brain samples.

Expression of various exon variants were examined in the single isoform mutant background as shown in Fig. S2f-i and line 256-265.

In wild type condition, we assume that the eighth variant of exon 9 (exon 9.8) is stochastically chosen upon alternative splicing. In contrast, the single isoform mutant, *dscam^3.31.8^*, lacks all variants of exon 9 except for 9.8. We next asked what happens to the expression pattern of exon 9.8 in this single variant mutant background (*dscam^3.31.8^/dscam^20^*). Surprisingly, mRNA for exon 9.8 was uniformly detected in the medulla NBs forming a circle encompassing the entire larval brain hemisphere (Fig. S2f, g), suggesting that exon 9.8 is always chosen in the absence of the other variants of exon 9. Consistently, mRNA for exon 9.1, 9.4, and 9.7 were not detected in the same mutant background (Fig. S2h, i and not shown). Taken together, these findings support our hypothesis that neurons of same lineage express similar Dscam1 isoforms.

Minor comments
Near the end of the introduction, Dscam is described as a gene putatively responsible for Down syndrome. There is no citation for this statement, and it might be better phrased as “significantly contributing to some of the phenotypes observed in Down syndrome” as other genes appear to have stronger overall effects.

We changed the phrasing as suggested and added citations #20 and #21 in line 92.

The introduction wonderfully puts the work into a broader context of neuronal organization. A formal definition of the medulla column should be given in the introduction to avoid confusion for the reader between the columns of differentiating neurons and the columns in the neuropil. Are the Ncad negative regions cell bodies or dendrites? The location of columns should be labeled in figure 1. Additional terms such as the medulla cortex should also be defined for clarity. 
The text was modified as follows in line 48-52. Importantly, the medulla columns are only found in the medulla neuropil, and the cell bodies of medulla neurons situated in the medulla cortex send axons and dendrites to the medulla neuropil to form the columns as added in Fig. 1a.

The medulla neurons make connections and form columnar units in the medulla neuropil ^8^. Note that medulla neurons, whose cell bodies are situated in the medulla cortex, project their neurites toward the medulla neuropil (Fig. 1a). The axons and dendrites of medulla neurons and the other neurons such as R7, R8, L1-5, *etc* form repetitive columnar units in the medulla neuropil.

Results: briefly explain why MARCM is used (to genetically label a small subset of cells).
The text was modified as follows in line 115-116:

using the MARCM technique in order to genetically label neurons of the same lineage (Fig. 1a, d).

Last sentence of second results paragraph – reminiscent may be a better word than luminescent.

Changed as suggested in line 133.

Full gene names should be presented once; e.g. homothorax/hth.
Full gene names were shown for homothorax (hth), brain-specific-homeobox (bsh), and drifter (drf) in line 67, 73, 76.

A similar pattern of mRNA expression in neuroblasts has been observed for the frazzled axon guidance receptor in the embryo (Joly et al. 2007 Dev. Biol. 301:542).

The following sentence and citation were added in line 306-307:
Similarly, a transcriptional regulator, Engrailed, regulates the expression of a guidance receptor, Frazzled, in NBs to control axon guidance during *Drosophila* embryonic development^31^.

Figure legends could generally include a little bit more detail to allow the reader to fully comprehend the figure. For example:
Fig. 2. State that in situ RT-PCR is being used.
Fig. 4. State the GAL4 driver used to make the clones.

Changed as suggested in line 649 and 679. Details were added to Figure legends.

Reviewer #3 (Remarks to the Author):
How large, partly repetitive arrays of neurons are properly wired up remains still largely unknown, even though several key pathways and principles have been identified in the past decades. One important part of the early decision on adhesion/repulsion is regulated by the adhesion pathway of the Down syndrome cell adhesion molecule (Dscam) gene. 
In the current manuscript Liu and colleagues investigate the role of DSCAM in the developing optic ganglia – more specifically in the medulla- of the fruit fly. First, they provide evidence that neurons from the same lineage project into distinct columns, suggesting “lineage-dependent repulsion”. They provide evidence that DSCAM is involved in the process as clones expressing the same isoform show disorganized projections. In an array of experiments, they build up a model in which the DSCAM is expressed in the NB, but the protein is required subsequently in the neuron; along the same lines they suggest that within a NB lineage, similar isoforms are expressed. 

The manuscript is intriguing and provides insight into the mechanisms that coordinate the structured development of the optic lobes. However, the core messages are not without problems, particularly since the claims of how systems appear to work are not quite intuitive. My main concern lies in the dynamic of RNA/protein expression.
While the DSCAM and Hth clones indeed show that the protein detection works, the RNA data is not as clear.

To improve the data of single exon PCR, we not only repeated exon 9 RT-PCR, but also added results of exon 4 and exon 6. We repeated in situ RT-PCR experiments and quantified the results as added in Fig. S1f-n and line 234-237.

We repeated the same experiment for 22 splice variants from exon 4 (4 variants), 6 (10 variants) and 9 (8 variants.; Material and methods). At least ten samples were observed for each variant, and we obtained essentially the same results as quantified in Fig. S1l-n, suggesting that an alternative splice variant is stochastically chosen.

Additionally, following experiments were added to verify the reliability of our in situ RT-PCR method. We tried control *Dscam1* RT-PCR in *Dscam1* null mutant clones, and found that it is abolished in mutant areas (Fig. S1b), suggesting that *in situ* RT-PCR specifically detects *Dscam1* mRNA (line 213-216).

To confirm the validity of our *in situ* RT-PCR technique, we examined *Dscam1* mRNA signal in clones homozygous for *Dscam^20^*, a null mutant of *Dscam1* (Fig. S1b). Compared with the control cells, signals for Dscam1 protein and *Dscam1* mRNA as visualized by *in situ* RT-PCR were abolished, suggesting that *in situ* RT-PCR specifically detects *Dscam1* mRNA.

Since Ncad is widely expressed in medulla neurons, we also visualized *Ncad* mRNA as a control by using *in situ* RT-PCR (Fig. S2c-e; line 217-223).

We also visualized *Ncad* mRNA by using *in situ* RT-PCR (Fig. S1c-e). Consistent with Ncad protein expression in medulla neurons, we observed strong *Ncad* mRNA signal in the inner region of the medulla cortex. Relatively uniform *Ncad* mRNA signal throughout the medulla strongly suggests that the sharp decrease of *Dscam1* mRNA signal inside the brain indeed recapitulates *Dscam1* expression (Fig. 2g-i). The gradual decrease of *Ncad* mRNA signal inside the brain might be caused by inefficient penetration of the reagents inside the brain samples.

Expression of various exon variants were examined in the single isoform mutant background as shown in Fig. S2f-i and line 256-265.

In wild type condition, we assume that the eighth variant of exon 9 (exon 9.8) is stochastically chosen upon alternative splicing. In contrast, the single isoform mutant, *dscam^3.31.8^*, lacks all variants of exon 9 except for 9.8. We next asked what happens to the expression pattern of exon 9.8 in this single variant mutant background (*dscam^3.31.8^/dscam^20^*). Surprisingly, mRNA for exon 9.8 was uniformly detected in the medulla NBs forming a circle encompassing the entire larval brain hemisphere (Fig. S2f, g), suggesting that exon 9.8 is always chosen in the absence of the other variants of exon 9. Consistently, mRNA for exon 9.1, 9.4, and 9.7 were not detected in the same mutant background (Fig. S2h, i and not shown). Taken together, these findings support our hypothesis that neurons of same lineage express similar Dscam1 isoforms.

Similarly, it is unclear how protein can be synthesized without RNA- at least at some stage of immature neurons there must be RNA present. 
I feel it would be important to further substantiate the claim/model, in particular molecular and/or genetic evidence.

As shown in Fig. 2g, i, signals for *Dscam1* mRNA are detectable in neurons. Dscam1 mRNA is transcribed in NEs and NBs, but it exists in neurons as summarized in Fig. 7. Importantly, Dscam1 mRNA signal in neurons was lost in *Dscam1* mutant clones as shown in Fig. S1b, suggesting that Dscam1 mRNA is detectable with our method. To avoid confusion, the following sentence was added in line 397-399:

The *Dscam1* mRNA and Dscam1 protein are then inherited by the daughter neurons of the NBs in a lineage dependent manner (Fig. 7a, b).

- It is intrinsically hard to have a NB specific experiment, and have the progeny express the protein. In the case of DSCAM it However, if indeed the RNA is only required in NBs one line of evidence could be gained by a neuron-specific manipulation. While this would be a negative result, showing that DSCAM expression is dispensable from neurons (what would be expected from the model) would support the notion.
Neuron-specific gene manipulation is very difficult, because even *elav-Gal4*, which is generally used as a neuron-specific Gal4 driver, is not completely specific to the medulla neurons. It is also expressed in NBs. Even if a neuron specific Gal4 driver is available, the result would be negative in any case because RNAi requires relatively long time to become effective.

- While the approach of using in situ PCR would be intrinsically elegant to distinguish different DSCAM isoforms the data displayed is not particularly clear. For instance, it is unclear if the approach allows to detect RNAs in deeper layers as overall the signal seems to be strong at the surface cell layer (maybe layer 2). Thus, the indirect quantification that is displayed may be faulty. 
Since much of the argumentation of the manuscript is based on the temporal dynamics of DSCAM RNA and protein a clarification of this point would be critical. For instance, control experiments with other markers (e.g. DPN, Lsc and more general ELAV) would support the notion of the authors.

Since Ncad is widely expressed in medulla neurons, we visualized *Ncad* mRNA as a general control in a deeper area of the brain by using *in situ* RT-PCRin line 217-223:

We also visualized *Ncad* mRNA by using *in situ* RT-PCR (Fig. S1c-e). Consistent with Ncad protein expression in medulla neurons, we observed strong *Ncad* mRNA signal in the inner region of the medulla cortex. Relatively uniform *Ncad* mRNA signal throughout the medulla strongly suggests that the sharp decrease of *Dscam1* mRNA signal inside the brain indeed recapitulates *Dscam1* expression (Fig. 2g-i).

- The experiment shown in figure 2h to test exon 9.1 RNA detection is not convincing. There appears to be signal on several NBs on one side of the brain. If so, this appears to be a domain-like expression and not in agreement with sparse stochasticity.

To improve the data of single exon PCR, we not only repeated exon 9 RT-PCR, but also added results of exon 4 and exon 6. We repeated in situ RT-PCR experiments and quantified the results as added in Fig. S1f-n and line 234-244.

We repeated the same experiment for 22 splice variants from exon 4 (4 variants), 6 (10 variants) and 9 (8 variants.; Material and methods). At least ten samples were observed for each variant, and we obtained essentially the same results as quantified in Fig. S1l-n, suggesting that an alternative splice variant is stochastically chosen.

We consider the following possibilities about the domain-like expression pattern of exon variants. Since *Dscam1* transcription starts in NEs, which quickly divide symmetrically, multiple NBs may share the same exon variant. Or, there might be additional mechanisms. We added the following sentences in line 238-244.

If the stochastic choise of an alternative exon solely occurs in NBs, a salt-and-pepper-like pattern should appear. Indeed, closer look at their expression patterns occasionally reveals a lack of *in situ* RT-PCR signals in an expression domain (Fig. S1i). However, this may be due to the cell cycle dependent changes in mRNA distribution. Since Dscam1 expression is initiated in NEs (Fig. 2d-f), which divide symmetrically, a group of NBs is supposed to share the same exon variant. Or, there might be unknown mechanisms that provide a bias on the choise of alternative exons in NEs.

The layout of figures and text are not well adherent; particularly the initial sections jump between figures and panels. A more logical layout would be more appealing and making it easier for a reader to follow the manuscript.

- Subsequent clonal analysis is clearer.
The layout of Figs. 2, 3, S1-2 were significantly modified so that the order of the figures are consistent with their order in the text.

- The beginning of the abstract jumps directly to the columnar organization without any introductory or explanatory sentence. It would be beneficial to provide some context.

Following sentences were added to the first paragraph of Introduction (line 25-28).

Neurons are the elementary units of the brain. Many neurons are precisely connected forming sophisticated neural circuits that enable our brain functions. On the other hand, columns are the higher-order morphological and functional units of the brain. A group of neurons gather to form individual columnar units, which are then precisely arranged to establish the brain.

**Reviewer comments, second version:**

Reviewer #1 (Remarks to the Author):

In general, I think the authors have done a good job of responding to the reviewer comments, and have significantly strengthened the manuscript. My only remaining question is why the quantification of the distance between neurites from the same radial unit appears so different in Fig. 1m (8.5 microns) and Fig. 5c (control clones, 50 microns). If these quantifications were done differently, the authors should explain what the difference was.

Jessica Treisman


Reviewer #2 (Remarks to the Author):

The extra detail on the RT-PCR is appreciated and I will be very interested to see the technique validated by other groups. The data quantification requested has been included. T-tests have been included in the figure legends, but significance not indicated on the actual graphs as is customary (Figs. 5g, 6e) and would be nice to add. Otherwise, the paper appears ready for publication.


Reviewer #3 (Remarks to the Author):

Many of the aspects raised have been addressed by the authors. However my main concern, which appears to be shared with the other reviewers remains. It is unclear to me if and how the newly introduced PCR based methodology for RNA detection is sufficiently sensitive and quantifiable.
The authors have now introduced a couple of additional controls, which in principle should be helping. However the data presented appear not clear either. The new data in supplementary figure 1 and 2 show no uniform signal making it hard to evaluate the issue.

While I'm positive regarding the relevance of the study, I remain doubtful about this part of the manuscript.
I would encourage the authors to seek for alternative more conventional proofs of their claims, including for instance insitu hybridisation variants.

**Author rebuttal, second version:**

Reviewer #1 (Remarks to the Author):
In general, I think the authors have done a good job of responding to the reviewer comments, and have significantly strengthened the manuscript. My only remaining question is why the quantification of the distance between neurites from the same radial unit appears so different in Fig. 1m (8.5 microns) and Fig. 5c (control clones, 50 microns). If these quantifications were done differently, the authors should explain what the difference was.

Jessica Treisman
In Fig. 5c, the clones contain multiple neurons (typically more than ten) and we measured the maximum distance among all of these neurons. In Fig. 1m, we only focused on a pair of two neurons, and the distance varies between 0 and 50 microns as shown in Fig. 1l (median and average of the distance are 5.78 and 8.50um, respectively). We added the following explanation in line 317.

'Note that we measured the largest distance among multiple neurons, which is greater than the average distance between two neurons (Fig. 1l).'

Reviewer #2 (Remarks to the Author):
The extra detail on the RT-PCR is appreciated and I will be very interested to see the technique validated by other groups.

Extra details were added to the in situ RT-PCR protocols (line 433-464).

The data quantification requested has been included. T-tests have been included in the figure legends, but significance not indicated on the actual graphs as is customary (Figs. 5g, 6e) and would be nice to add. Otherwise, the paper appears ready for publication.

P-values were added to Figs. 5g, 6e legends (line 786, 803).

Reviewer #3 (Remarks to the Author):
Many of the aspects raised have been addressed by the authors. However my main concern, which appears to be shared with the other reviewers remains. It is unclear to me if and how the newly introduced PCR based methodology for RNA detection is sufficiently sensitive and quantifiable.
The authors have now introduced a couple of additional controls, which in principle should be helping. However the data presented appear not clear either. The new data in supplementary figure 1 and 2 show no uniform signal making it hard to evaluate the issue.
While I'm positive regarding the relevance of the study, I remain doubtful about this part of the manuscript. 
I would encourage the authors to seek for alternative more conventional proofs of their claims, including for instance insitu hybridisation variants.

We have already added negative and positive controls for *in situ* RT-PCR in Supplementary Figs. 1, 2. *in situ* RT-PCR is far more sensitive compared with regular *in situ* hybridization protocols.

**Reviewer comments, third version:**

**Author rebuttal, third version:**
